# Supplementary material for: Digital measurement of SARS-CoV-2 transmission risk from 7 million contacts
Source: Nature. 2023 Dec 20;626(7997):145–50. doi: 10.1038/s41586-023-06952-2 (PMC10830410; doi:10.1038/s41586-023-06952-2)
Supplement: Supplementary file 1 — Supplementary Methods, Discussion, Tables 1–5, Figs. 1–13 and pseudocode for processing raw app data. [file 41586_2023_6952_MOESM1_ESM.pdf]

nature portfolio

https://doi.org/10.1038/s41586-023-06952-2

Supplementary information

Digital measurement of SARS-CoV-2 transmission risk from 7 million contacts

In the format provided by the authors and unedited

Supplementary Information
Digital measurement of SARS-CoV-2 transmission risk from 7 million contacts

Luca Ferretti, Chris Wymant, James Petrie, Daphne Tsallis, Michelle Kendall, Alice Ledda, Francesco Di Lauro, Adam Fowler, Andrea Di Francia, Jasmina Panovska-Griffiths, Lucie Abeler-Dörner, Marcos Charalambides, Mark Briers, Christophe Fraser

Contents

1 Supplementary Methods 2
- 1.1 Risk scoring for the NHS COVID-19 app . . . 2
- 1.2 Exposure data . . . 3
  - 1.2.1 The raw data . . . 3
  - 1.2.2 How we processed the raw data . . . 4
  - 1.2.3 Schematic example of data flow . . . 6
- 1.3 Accuracy of the correspondence between exposure groups and case-contact pairs . . . 7
  - 1.3.1 Upper bound for the fraction of incorrect groupings . . . 9
  - 1.3.2 Single versus multiple index cases . . . 10
- 1.4 Empirical estimation of individuals' probability of testing positive from summary statistics . . . 14
- 1.5 Theoretical expectations from microbial risk assessment . . . 15
- 1.6 Statistical modelling of the per-exposure-window probability of transmission . . . 20
  - 1.6.1 The main model . . . 20
  - 1.6.2 Incorporating ascertainment bias and risk heterogeneity . . . 21
- 1.7 Distributions of predictors . . . 25
- 1.8 Predicting which contacts report positive tests . . . 26

2 Supplementary Discussion 27
- 2.1 Predictive power of app-measured scores for classification of contacts 27
- 2.2 Optimising interventions for contacts with intermediate risk of infection 30
  - 2.2.1 Amber notification and testing of contacts . . . 30
  - 2.2.2 Optimal strategy for post-exposure prophylaxis . . . 31

3 Pseudocode for processing raw app data 35

1 Supplementary Methods

1.1 Risk scoring for the NHS COVID-19 app

The NHS COVID-19 app ('the app' henceforth) uses the Google-Apple Exposure Notification (GAEN) framework to assess the risk score of exposures to confirmed COVID-19 cases. Each prolonged exposure event is split into separate exposure windows, each lasting at most 30 minutes. Risk is computed separately for each window using three pieces of information:

1. proximity between individuals, ρ: the GAEN framework measures Bluetooth Low Energy (BLE) signal attenuation and uses it as a proxy for distance between mobile phones. The attenuation is measured every time a key is received, which is typically every few minutes. The app processes the time series of attenuation values through an Unscented Kalman Filter that estimates the posterior trajectory of the distance between individuals (Lovett et al., 2020) for more accurate distance estimation. The mean posterior distance is then weighted by the duration of the time between keys and averaged over the whole exposure.

2. duration of exposure, d: the app records the total duration between the first and the last keys received during the 30-minute window.

3. infectiousness of the index case, χ: the GAEN framework encodes three levels of infectiousness, determined from the only relevant information available to the app which is the day of the exposure relative to the day of the index case's onset of symptoms (or positive test result if symptom onset was not reported). The infectiousness for a given number of days from onset of symptoms to exposure is obtained by discretisation of the continuous infectiousness curve in (Ferretti et al., 2020; Fraser et al., 2020). In agreement with UK guidelines on contact tracing, all exposures more than 2 days before onset of symptoms were deemed not risky (i.e. low risk). The resulting infectiousness table is:

Day of exposure relative to index's onset of symptoms:	≤ -3	-2 *to* +3	+4 *to* +9	$\geq +10$
Infectiousness:	"low"	"high"	"standard"	"low"
Coefficient χ:	0	2.5	1	0

The overall scaling of the risk score for a window (and thus of each of its three multiplicative components) is arbitrary: action is only taken depending on its size relative to the threshold for notification, which can be scaled correspondingly. For ease of interpretation, we normalised all risk scores by the risk score for a 15-minute encounter with an infected individual of standard infectiousness at 2 metres' distance. With this normalisation, the risk score r is

$$r = \frac{s(\rho) \cdot d \cdot \chi}{s(2\,\mathrm{m}) \cdot 15\,\mathrm{min} \cdot 1} = \frac{s(\rho) \cdot d \cdot \chi}{3.75\,\mathrm{min}} \tag{1}$$

where the proximity score that defines the dependence of the risk of transmission with distance is constant below 1 metre and decreases quadratically for distances above 1 metre (Briers et al., 2020; Fraser et al., 2020):

$$s(\rho) = \begin{cases} 1\,\mathrm{m}^2/\rho^2 & \text{if } \rho > 1\,\mathrm{m} \\ 1 & \text{if } \rho \leq 1\,\mathrm{m} \end{cases} \tag{2}$$

With this normalisation, the threshold for notification used by the app throughout the period studied was 10/9, and the maximum possible score was 20.

1.2 Exposure data

1.2.1 The raw data

As we described in greater detail previously (Wymant et al., 2021 and Kendall et al., 2023) each installation of the app on a mobile device sends a single 'analytics packet' daily to the central server with a small amount of data. This includes the following four data fields:

1. postcode district (the first half of the postcode, user-declared). To further preserve anonymity, when postcode districts have a population of less than 5000, data is automatically amalgamated with nearby districts.

2. Lower-tier local authority (LTLA; user-declared if ambiguous from the postcode district).

3. Operating system version

4. Device model (e.g. 'iPhone X').

Henceforth we refer to the four variables above as the 'individual-level' characteristics or data, because they are expected to remain constant for a given individual over the time scale of one round of exposure, contact tracing and testing. For other data fields this is not true, for example those recording technical functioning of the app, and the data field indicating whether or not the user was notified that day of a risky exposure. These analytics packets contain no sensitive or identifying information, and are approved and publicly listed by the Information Commissioner (https://www.gov.uk/government/publications/nhs-covid-19-app-privacy-information).

An additional type of data is available for the app, made up of 'events packets', which we describe below. For each exposure window with a risk exceeding the threshold for notification, an event packet is sent to the central server. We denote the set of all recorded exposure windows by E (or E_X when we define a subset of windows satisfying the criterion X, see later). We use e to index a particular exposure window (or, with a slight abuse of notation, the event packet recording data about that exposure window). For each exposure window $e \in E$, the event packet contains the aforementioned individual-level data fields 1-4, and the following data specific to the window:

1. risk score r_e (greater than the threshold $10/9 \approx 1.1$ for all event packets in our analysis);

2. data on attenuation and duration of exposure. The smartphone records all BLE scans for nearby devices during the window: for each BLE scan (typically one every 2-3 minutes), the average and minimum attenuation in dB of the BLE signal between smartphones, as well as the number of seconds elapsed since the previous scan, are included in the packet;

3. infectiousness category of the index case;

4. duration d_e;

5. exposure date t_e;

6. date on which the user was notified of their risky exposure, n_e. The true notification date is not reported, but it should coincide with the date the event packet was received by the backend.

As described in Methods, if an individual reports a positive test in the app during the 'observation interval'—starting with their being notified, ending with 14 days since the exposure—the event packets that triggered the original notification are retrieved once more and sent to the central server. Henceforth we refer to 'reporting a positive test through the app during the observation interval' as 'testing positive' for brevity, though it is important to remember that individuals with this outcome are a subset of all infected individuals due to incomplete case ascertainment.

Data collection was performed using RAthena (v2.6.1) queries of the app database. In a preliminary step, we performed a basic cleaning of the data by deduplicating identical packets received at different times (collisions in packet content are extremely unlikely to occur by chance, therefore it is natural to assume that these packets have been sent repeatedly by the same smartphone) and filtering out packets with timing inconsistent with app specifications (e.g. time from exposures to notification of more than 14 days) or without user-declared LTLA.

1.2.2 How we processed the raw data

Event packets do not contain a unique identifier for the notified individual whose app sent them. Therefore to determine whether a given event packet sent at the notification stage was later sent again at the test-positive stage by the same individual, we checked for the existence of a later event packet that was identical to the previous event packet in every field of data (the individual-level characteristics 1-4 and the exposure-window characteristics 1-6 above) except for the field indicating whether the packet was sent at the notification stage or the test-positive stage. If such a matching packet was found, we assumed that the individual tested positive; if it was not found, we assumed that the individual did not test positive.

The assumption above allows the outcome of testing positive or not to be associated to each exposure window, which we encoded in a binary variable $\pi_e \in \{1, 0\}$ for exposure window e (1 denoting test positive, 0 otherwise).

The complete exposure of a given contact to their associated index case is in general recorded in multiple exposure windows, which are not linked to each other in the raw data due to the absence of a unique identifier for each app user. In order to reconstruct the complete exposure for each contact, we assumed that when exactly one individual with a specific combination of individual-level characteristics was notified on a given date (as indicated by the analytics packets, counting those for notified users grouped by date and by the individual-level characteristics 1-4), then all event packets with those individual-level characteristics on that date could be grouped together as from a single putative individual, as opposed to several individuals with matching characteristics. For a given notified individual $i \in I$, defined by a set of individual-level characteristics and a notification date, the set of grouped exposures is denoted by E_i. Exposures that cannot be assigned unambiguously to a single individual are discarded.

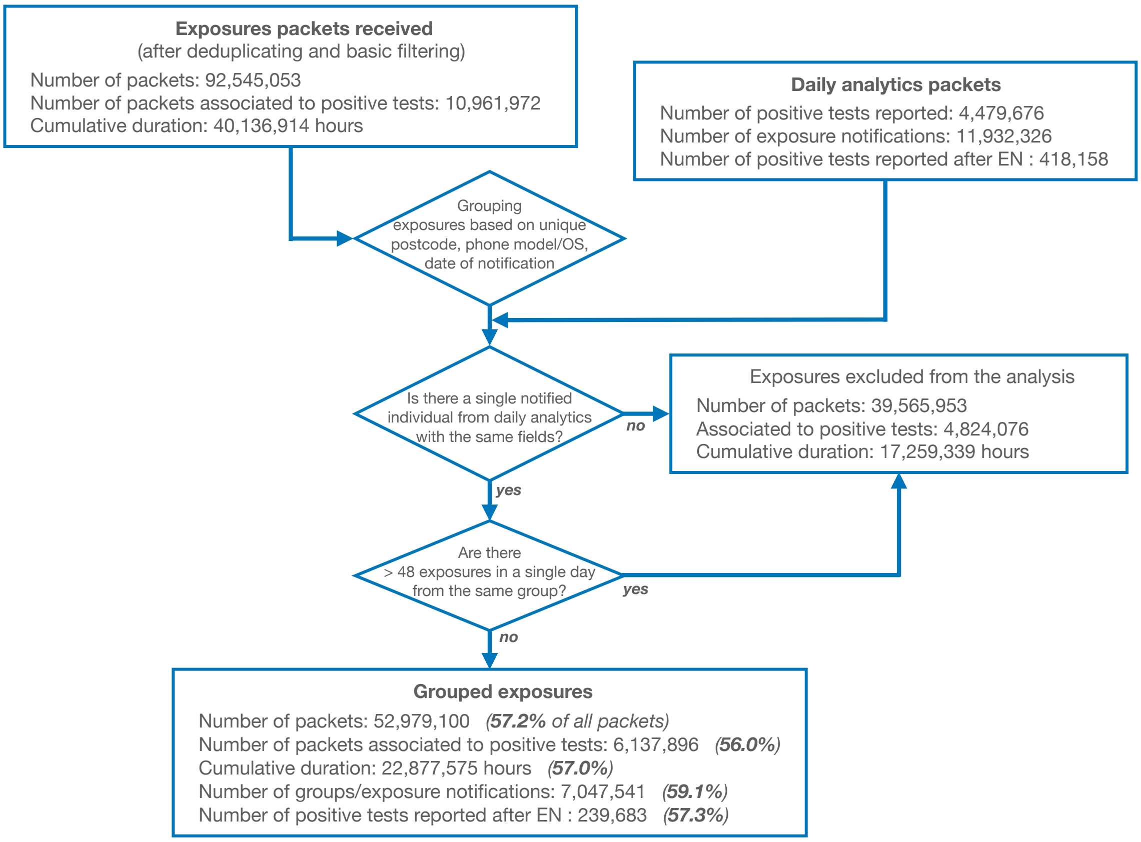

Figure S1: Flowchart of the available data and grouping/filtering process.

	**All event packets (before grouping)**	**Grouped events packets**	**Grouped event packets for individuals reporting positive**
**Number of packets**	92,545,053	52,979,100 (57% of left value)	6,137,896
**Number of packets associated to positive test**	10,961,972 (11.8% of above value)	6,137,896 (56% of left value; 11.6% of above value)	6,137,896
**Cumulative duration (hours)**	40,136,914	22,877,575 hours (57% of left value)	2,670,167 (11.7% of left value)
**Mean duration per window (minutes)**	26	26	26
**Number of contacts**	12,013,981	7,047,541 (59% of left value)	239,683 (3.4% of left value)
**Mean duration per contact**	3 hours 20 minutes	3 hours 15 minutes	11 hours 8 minutes
**Mean number of packets per contact**	7.76	7.52	25.61

Table S1: Summary of the event packets data at different stages.

The flowchart of the process is illustrated in Figure S1 and the summary statistics are reported in Table S1.

Included with the paper is a supplementary file of R code illustrating unambiguously the set of processing steps we applied to the raw data to arrive at the form of the dataset analysed here.

1.2.3 Schematic example of data flow

- Day 1: app users Alice and Bob are proximate for most of the day. By proximate we mean specifically that they were sufficiently close to exchange BLE signals. The signal sent by Alice's device to Bob's encodes the 'key' currently used by Alice (and vice versa). Keys are unique, anonymised, randomly assigned codes that are regularly changed to prevent individual identification.
- Day 2: Alice and Bob are proximate from 10:45 to 12:00.
- Day 3: Alice and Bob are proximate from 10:00 to 10:30.
- Day 4: Alice develops symptoms.
- Day 5:
  - Alice reports a positive test in the app.
  - Alice consents to key sharing, meaning that her app sends to the central server the set of keys that her app has recently used to identify itself. The central server updates its list of all such keys shared by cases. NB this central list is not of the keys of other apps encountered by cases, by construction of the 'decentralised' system of key sharing: the keys stored centrally identify only cases, not contacts.
  - Bob's app compares two lists of keys: the central list of keys of cases, and its own list of keys for other devices recently encountered by Bob which is stored only on his device. (This comparison is made regularly by all devices running the app. If the two lists have no key in common, no further action is taken.) Bob's app recognises some overlap in the lists, which implies that he was recently proximate to a case. Specifically, keys recently used by Alice are in both lists.
  - Bob's app analyses the data for the exposure to Alice, recorded in separate exposure windows of 30 minutes:
    * Exposure windows from day 1 are all three days before Alice's onset of symptoms; for these, her infectiousness is classed as 'low' with χ coefficient zero, so the risk scores are all zero.
    * The first exposure window from day 2 is 10:45 to 11:15. The inferred distance ρ is large (the proximity score $s(\rho)$ is small), resulting in a risk score for this window that is below the threshold for notification.
    * The second exposure window from day 2 is 11:15 to 11:45. The risk score is over the threshold: this is the first risky window.
    * The third exposure window from day 2 is 11:45 to 12:00. The risk score is over the threshold: this is the second risky window. (The duration was only 15 minutes but the inferred distance ρ is small enough to compensate.)

        * The only exposure window from day 3 is 10:00 to 10:30. The risk score is over the threshold: this is the third risky window.

    - Bob's app has thus found three risky windows; one would be sufficient for a notification, and so Bob is notified of a recent risky exposure.
    - Bob's app sends three events packets to the central server: one for each risky window.
    - The central server receives three events packets. The three packets have no unique ID for which mobile device sent them; however, they have common values for individual-level characteristics (postcode district, LTLA, operating system version, device model) and a common inferred date of notification (the date they were received by the central server, i.e. day 5). The three packets differ in the details of the exposure because they record data about three different exposure windows (though by construction all of them are *risky* exposure windows, and all are for an exposure to the same index case). The packets contain no information about Alice except whether her infectiousness was 'high' or 'standard', which was used to determine Bob's risk.
    - At midnight Bob's app sends an analytics packet to the central server, as do all correctly functioning apps every day. The packet includes the aforementioned individual-level characteristics and indicates that Bob was notified today.

- Day 7: Bob reports a positive test in his app. Bob's app resends to the central server the same three event packets as when Bob was notified, except for the data field that indicates that this is the test-positive stage rather than the notification stage.
- At any later date, we analyse the data:
    - For every notification-stage event packet e we check whether a later event packet exists that is identical except for being at the test-positive stage. If so, we assign $\pi_e = 1$ to the packet to indicate the test positive outcome.
    - Grouping analytics packets by individual-level characteristics, date, and whether the user was notified on that date, we observe only one such packet like Bob's on the date they were notified, i.e. no other individual with identical individual-level characteristics was notified on day 5.
    - We therefore assume that the three events packets with those individual-level characteristics received on day 5 came from the same notified individual, as opposed to e.g. three different individuals with matching characteristics who were notified on the same date. Reassuringly all three packets have the same value of π_e which is consistent with their being from a single individual. The three packets have $\pi_e = 1$, and so we assign $\pi_i = 1$ for the individual i.e. they tested positive.

1.3 Accuracy of the correspondence between exposure groups and case-contact pairs

Grouping exposures results in a loss of about 42.8% of all packets, but the grouping process does not appear to significantly affect any of the estimates of interest in this

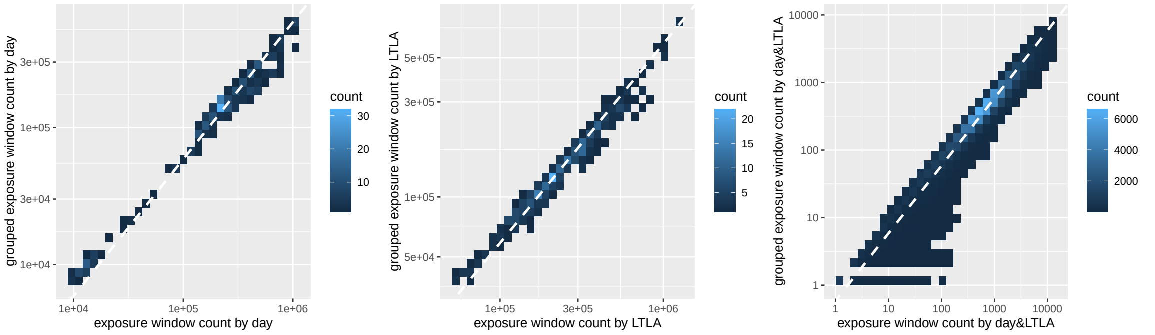

Figure S2: Two-dimensional histograms of the number of event packets (all packets on the x axis; packets that could be grouped as from a single contact on the y axis) by day of exposure (left), lower-tier local authority (LTLA) (center) and combinations of day and LTLA (right). The histogram is produced by iterating through every combination of date and/or LTLA; for each one, counting the number event packets with an exposure on that date and/or for that LTLA, defining x, and counting which of those packets could be grouped as from a single contact, defining y (which must be equal to or less than x), then updating the overall count for this value of x and y by 1, then proceeding to the next combination of date and LTLA. The dashed line shows the expected trend $y = m\,x$ if the grouped fraction would be always equal to m $=$ *total number of grouped packets / total number of packets*, i.e. to the dataset-mean probability of a packet being grouped. If our success rate in grouping packets as from a single contact was unbiased with respect to time and space, we would expect each combination of date and/or LTLA to have a y value drawn randomly from a binomial distribution with x trials and probability m, i.e. counts would cluster close to the dashed line.

paper. There is little difference in the average duration of exposure (3 hours and 25 minutes for all exposures versus 3 hours and 27 minutes for the grouped ones), in the fraction of exposures associated to a positive test (11.6% of all exposures versus 12.3% of the grouped ones), or in the estimate of the fraction of contacts reporting a positive test (3.5% from daily analytics data versus 3.6% from grouped exposures). Grouping exposures may introduce biases in the distribution of exposures in time and space. However, the additional noise does not seem to lead to strong distortions of the spatiotemporal distribution: the Pearson correlation between the number of all exposures versus grouped exposures by day of notification is $r = 0.97$, the correlation by LTLA is $r = 0.98$, the correlation by day and LTLA is $r = 0.95$ (see Figure S2). Hence, these biases are unlikely to have any relevant impact on our analysis.

If not all daily analytics packets would be received, some of the groups may actually correspond to multiple contacts whose exposures have been incorrectly grouped. In this case, the combined validity of the assumptions described in section 1.2.2 could be tested using the fact that a single individual has a binary outcome: they either test positive or they do not. Therefore, if we assumed correctly in grouping multiple notification-stage event packets together as from the same individual i, either all of these packets or none of them should be observed again at the test-positive stage: either all of the $e \in E_i$ should have $\pi_e = 1$ or all should have $\pi_e = 0$. Observing some but not all of the packets again at the test-positive stage—a mixture of 0s and 1s for the individual's π_e values—implies at least one of two problems. Either the event packets in this group were not sent by a single individual as assumed (with at least one individual testing positive and at least one other individual not), or some

packets were missing at the test-positive stage (which would jeopardise the interpretation of the absence of such packets as an indication that the notified individual did not test positive). Technical issues such as packet losses, duplications and delays can result in such problems. Reassuringly, out of all putative individuals (i.e. separately grouped event packets) with at least two event packets, for 99.25% of them either all notification-stage packets were observed again at test-positive stage (4.3%) or none of them were (95%).

To classify the other 0.75% of these individuals—those with some but not all of their notification-stage event packets detected at the test-positive stage—we assumed they tested positive if and only if at least 2 of the notification-stage event packets were observed again at test-positive stage. This assignment of test positivity for individual i was encoded in the boolean variable π_i, i.e. we defined

$$\pi_i = \begin{cases} 1 & \text{if } \sum_{e \in E_i} \pi_e \geq \min(2, |E_i|) \\ 0 & \text{otherwise} \end{cases} \tag{3}$$

1.3.1 Upper bound for the fraction of incorrect groupings

An approximate upper bound for the fraction of incorrectly grouped exposure windows can be obtained as follows. In principle, for each contact we should either observe all of their exposure windows again at test-positive stage or observe none of them again. For the purpose of deriving this upper bound, assume that the fraction of incorrectly grouped windows is small, and ignore individuals with 3 or more exposure windows (given how skewed the distribution over contacts is towards short exposures). Suppose there are N true individuals in total. Let ω be the fraction of individuals who report exactly two windows of exposure. Let p_1 be the positivity rate for individuals who report exactly one window, and p_2 the positivity rate for individuals who report exactly two windows. A fraction f of the $N(1-\omega)$ individuals who report one window of exposure will be incorrectly grouped together to form one putative individual with two windows of exposures each, for a total of $N(1-\omega)/2$ incorrect putative individuals. This is assumed to happen randomly and independently of their positivity. Similarly, some true individuals who have two windows of exposures will be grouped with other individuals to form incorrect putative individuals who report 3 or more exposure windows, that are excluded from this analysis. The contribution of these erroneous grouping will be of order $O(f)$ and can be ignored at the lowest order. In Figure S3, all these groups are listed, and equations for the five unknowns variables N, ω, f, p_1, and p_2 can be derived from the following data:

- n_1^+, the number of putative individuals with exactly one window, that window being positive ($\pi_e = 1$)
- n_1^-, the number of putative individuals with exactly one window, that window being negative ($\pi_e = 0$)
- n_2^{++}, the number of putative individuals with exactly two windows, both positive
- n_2^{--}, the number of putative individuals with exactly two windows, both negative
- n_2^{+-}, the number of putative individuals with exactly two windows, one positive and one negative

which lead to

$$\begin{cases} n_1^+ = N(1-\omega)p_1(1-f) \\ n_1^- = N(1-\omega)(1-p_1)(1-f) \\ n_2^{++} = N\omega p_2 + N(1-\omega)f\dfrac{p_1^2}{2} \\ n_2^{+-} = N(1-\omega)fp_1(1-p_1) \\ n_2^{--} = N\omega(1-p_2) + \dfrac{1}{2}N(1-\omega)f(1-p_1)^2 \end{cases}$$

It is also useful to define $n_1 = n_1^+ + n_1^- = N(1-\omega)f$, and $n_2 = n_2^{++} + n_2^{+-} + n_2^{--} = N - \frac{1}{2}N(1-\omega)f$. The solution is found as follows: find p_1 by dividing the first equation by the sum of the first two, find f by dividing the second equation by the fourth one, find ω by dividing the sum of the last three equations by the sum of the first two, derive N from the sum of the last three equations, and find p_2 from any equation involving it. This yields

$$\begin{cases} p_1 = \dfrac{n_1^+}{n_1} \\ f = \dfrac{n_2^{+-}}{n_1^- p_1 + n_2^{+-}} \\ \omega = 1 - \dfrac{1}{\frac{n_2}{n_1}(1-f) - \frac{f}{2} + 1} \\ N = \dfrac{n_2}{\omega + \frac{1-\omega}{2}f} \\ p_2 = \dfrac{n_2^{++} - N(1-\omega)f\frac{p_1^2}{2}}{n\omega} \end{cases}$$

Substituting in the counts from event packet data, the solution to this system yields $N = 6365000$, $p_1 = 0.018$, $p_2 = 0.023$, $\omega = 0.24$, $f = 0.077$, from which the fraction of misclassified grouped individuals becomes $\frac{(1-w)\frac{f}{2}}{w+(1-w)\frac{f}{2}} = 0.11$.

Therefore we can estimate that incorrectly grouped exposures are found in at most 11% of all individuals with multiple exposures, suggesting that our grouping approach provides a correct reconstruction for more than 89% of these individuals (and more than 94% of all individuals).

3,385,376 putative individuals (i.e. 48% of all putative individuals) had only a single event packet and so were unambiguously classified as testing positive or not based on whether that single event packet was observed again at the test-positive stage or not.

1.3.2 Single versus multiple index cases

Each installation of the NHS COVID-19 app on a mobile device regularly accessed the central server to check whether the central list of keys for index cases contains any keys stored locally on this device, i.e. whether this app user has had some level of exposure to a newly reported index case. It is possible that in the space of time between checking once and checking again, two index cases to which this app user has had risky exposure both report a positive test. Then this app user would become a notified contact due to two index cases rather than one, and their set of event packets

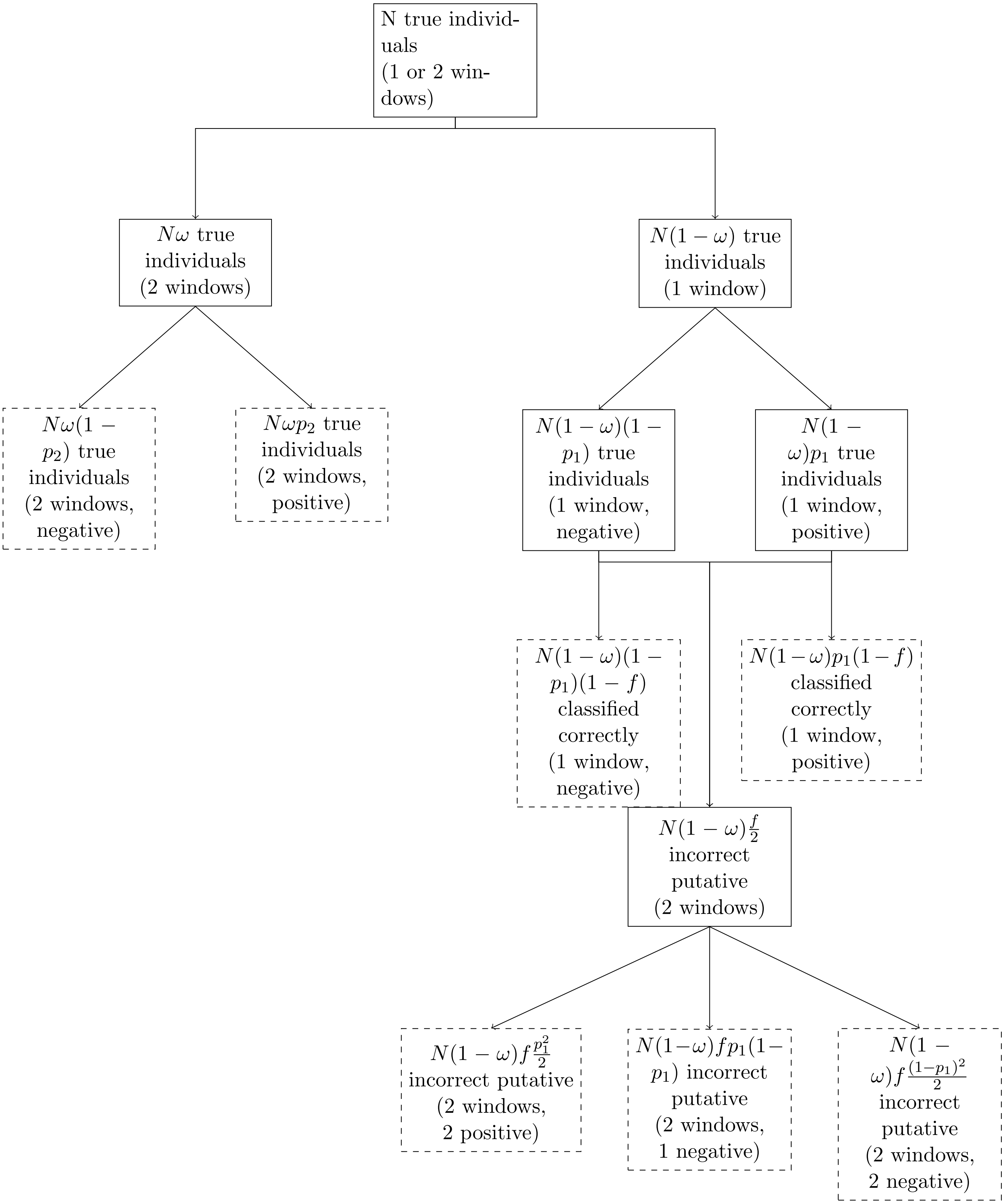

Figure S3: Schematics of all classification possibilities for grouping together up to two individuals. The dashed rectangles indicate that the term is included in the system of equations to derive the upper bound error for incorrect classification.

would record the history of exposures to two index cases rather than one. (If another index case reports a positive test after the exposed individual has been notified, this has no effect for the duration of the quarantine period.) We cannot distinguish between the scenarios of one or multiple index cases triggering notification, because the only information about the index case available to the contact's app (and then present in the event packets) is the index cases' binary infectiousness level—standard or high—at the time of exposure. However, in this section we explain why the scenario of multiple index cases simultaneously triggering a single notification is expected to be extremely rare. The short answer is the high frequency of the app checking for new index cases – roughly once per two hours.

When active, each NHS COVID-19 app instance retrieves keys corresponding to index cases from the central server with an approximate frequency of once every two hours. In case of multiple exposures, only the exposures associated with the first batch of retrieved keys trigger a notification and are reported to the central server. In order for the exposures reported to the central server to come from two or more index cases in contact with the same notified individual, these index cases must have uploaded their test results roughly at the same time, i.e. the maximum temporal distance between the time the first two index cases share their keys should be typically less than two hours.

We derived a simple model to estimate the probability that a contact would be notified by two putative index cases simultaneously. We modelled each infected individual who reports their test result on the app as a realisation of a stochastic process, with four possible events: infection at time t_0, symptoms onset at time $t_1 > t_0$ with probability distribution $p_S(t_1|t_0)$, testing at time $t_2 > t_1$ with probability distribution $p_T(t_2|t_1, t_0)$, and reporting a positive test through the app at time $T > t_2$ with probability density $p_R(T|t_2, t_1, t_0)$. We modelled this process as time homogeneous (all times can be shifted by the same amount with no effect), with each event time probability depending only on the time since the previous event. One can therefore set $t_0 = 0$ for convenience without loss of generality, and the aforementioned conditional probability distributions simply to $p_S(t_1)$, $p_T(t_2 - t_1)$, $p_R(T - t_2)$ respectively. Define $p(T)$ as the probability density for a time of reporting T, conditional on infection at time $t_0 = 0$ but with unobserved times t_1 and t_2 (which we therefore marginalise over):

$$p(T) = \int_{t_1=0}^{T} \int_{t_2=t_1}^{T} p_R(T - t_2) p_T(t_2 - t_1) p_S(t_1) dt_2 dt_1 \tag{4}$$

We modelled the delay from symptoms to test as lognormally distributed with mean 5.42 days and standard deviation 2.7 days (McAloon et al, BMJ Open 2020), the delay from booking a test to receiving results as gamma distributed with mean 1.5 days with standard deviation 0.5 days (in January 2021, https://www.gov.uk/government/collections/nhs-test-and-trace-statistics-england-weekly-reports), and the delay between symptoms and booking a test as gamma distributed mean 1.5 days and standard deviation 1.5 days (https://www.gov.uk/guidance/coronavirus-covid-19-getting-tested). In Figure S4 we show the reported the delay distributions from infection to symptoms onset, from symptoms onset to updating the results on the app, and the whole delay distribution $p(T)$.

We modelled two putative index cases as infected at time t_0 and $t_0 + \delta t$, both of whom went on to expose the same contact at some later time. Conditional on both

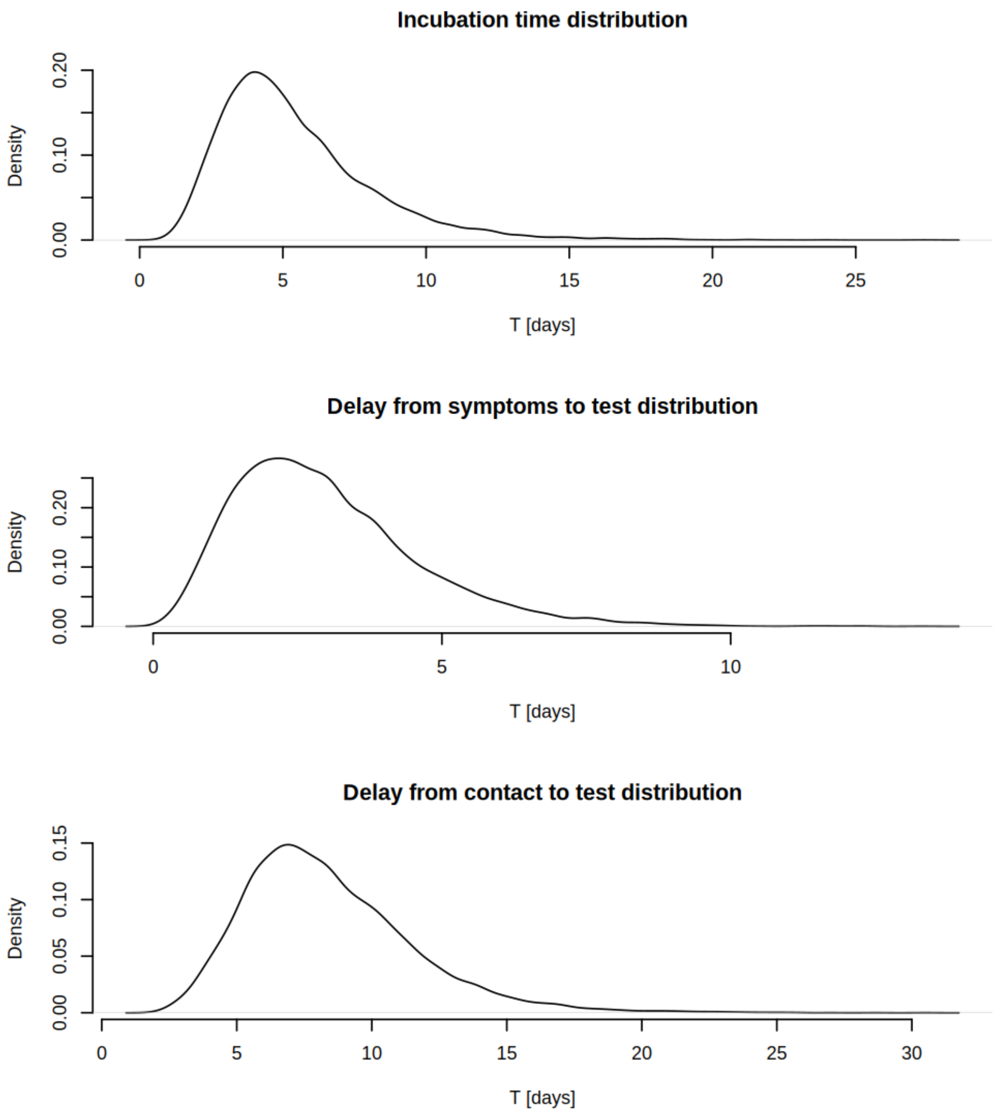

Figure S4: Delay distribution considered in modelling the probability of grouping exposures when multiple index cases expose the same notified individual at the same time.

of these individuals reporting their positive tests through the app and consenting to key sharing *at some point*, the probability that they do this during the same two-hour window can be estimated by simulating many pairs of individuals and counting how often they are found reporting the test within the same 2-hour retrieval window. When more than two putative index cases are considered, one need only consider the time difference between the first two reporting a test, as the app sends a notification as soon as it receives the first prompt from any index case. In Table S2 we report these estimated probabilities, for exposure to 2, 3, 4, and 5 contacts/index cases, assuming that each contact was infected at a random time distributed as a Gaussian with given standard deviation.

Note that the probabilities derived above, which are already small, are conditional upon (a) two (or more) putative index cases being infected, (b) both then having exposure to the same contact whose notification we are considering, (c) both choosing to test, (d) both report their test through the app, (e) both consenting to key sharing.

Table S2: Probability that a notification would have been caused by exposures to two or more infected app users at the same time. Results obtained from 1 million simulations for each scenario.

Delay distribution for δt (hours)	Probability if exposure to ... active app users			
	2 infected	3 infected	4 infected	5 infected
SD = 0	0.00887	0.01398	0.01858	0.02197
SD = 12	0.00851	0.01392	0.01803	0.02068
SD = 24	0.00751	0.01286	0.01609	0.01911
SD = 48	0.0067	0.01041	0.013	0.01526
SD = 72	0.00582	0.00899	0.01062	0.0123

Conditional on all these events happening, we calculated the probability for the final event being synchronised between the two index cases. However, to calculate the overall probability of this scenario we should also include the probabilities of these events happening, making the overall probability even smaller. The most likely scenario where multiple exposures happen is the exposed app user in question meeting multiple social acquaintances at the same event, with one of these acquaintances being infected and transmitting to more than one of the others at the event, e.g. in a super-spreading event. The probability of a superspreading event involving 2 or more active app users can be approximately estimated from the information of Kendall et al., 2023. In a social event with N people one expects $M = \alpha N$ having the app installed and active, with $\alpha \sim 0.2 - 0.3$ in the period under study. Of these, only a proportion $Y = \chi M$, with $\chi \sim 0.5$, would give consent to share the key to the app, conditional on testing positive and reporting it in the app. Taken altogether, this means that roughly only $10 - 15\%$ of the individuals infected in a superspreading event would be active app users, and therefore potentially able to act as multiple index cases for the same notified contact. This means that to be exposed to e.g. 2 active app users who were infected at the same event and decided to test and share their keys, the overall number of individuals infected during the same superspreading event should have been of the order of 7-10. One must also consider the fraction of all transmission events in which multiple people were infected by the same source; in a period in which the reproduction number R was typically close to 1, this fraction is small, even with some overdispersion being observed for SARS-CoV-2.

In practice, we can safely assume that almost all exposure groups correspond to a single index case.

1.4 Empirical estimation of individuals' probability of testing positive from summary statistics

For our simple empirical estimates linking exposure data to the test-positive outcome, we summarised each individual's set of exposure windows, $E_i = \{e_{i,1}, \dots e_{i,|E_i|}\}$, by

the following summary statistics as risk predictors:

$$\text{maximum risk score } r_i^{\max} = \max_{e \in E_i}(r_e) \tag{5}$$

$$\text{cumulative risk score } r_i^{\text{cum}} = \sum_{e \in E_i} r_e \tag{6}$$

$$\text{cumulative duration } d_i = \sum_{e \in E_i} d_e \tag{7}$$

$$\text{mean risk score } \bar{r}_i = \frac{1}{|E_i|} \sum_{e \in E_i} r_e \tag{8}$$

We estimated the probability $P_p(X)$ that an individual satisfying a condition X would test positive. The conditions we considered were whether or not the summary statistics defined in equations 5-8 fell in particular intervals (e.g. a cumulative duration of less than one hour). We denote by I_X the subset of individuals satisfying by the condition X. The number of such individuals is $|I_X|$ and the number of them testing positive is $\sum_{i \in I_X} \pi_i$, so the maximum-likelihood estimate of the probability of testing positive given condition X is trivially

$$\hat{P}_p(X) = \frac{1}{|I_X|} \sum_{i \in I_X} \pi_i \tag{9}$$

and we derived the associated confidence intervals from the associated binomial distribution (observing $\sum_{i \in I_X} \pi_i$ successes out of $|I_X|$ trials).

We estimated how P_p varied with the summary statistic $r^{\max}$. We calculated the value of P_p for the reference risk $r = 1$, as it is easily interpretable: a risk score of 1 is the threshold for manual tracing, namely 2 metres for 15 minutes with standard infectiousness. Calculating P_p for $r = 1$ required extrapolation because the app's threshold for notifying an individual of risky exposure was $10/9 \approx 1.1$, and our dataset comprised only notified individuals. Therefore we first computed a binned version of $P_p(r)$ from contacts with a single exposure window only, then we considered only values of $r < 3$ and performed a quadratic regression extrapolation of $P_p(r)$ to $r = 1$, denoting it by $\hat{P}_p(r = 1)$.

To correct for background risk, we subtracted the appropriate estimates for background infection rates (i.e. the background risk for all app users b_i and the maximum-likelihood estimate B_i^{ML} presented in the next section) from π_i. The maximum-likelihood background risk correction was $\pi_i^{\text{corrected}} = \pi_i - B_i^{ML}$, while the "full" background risk correction would be $\pi_i^{\text{corrected}} = \pi_i - b_i$. The latter is not used as it would be an overcorrection, as shown in Figure **??**.

The empirical correlations between some of these quantities in our dataset are presented in Tables S3, S4.

1.5 Theoretical expectations from microbial risk assessment

We first outline some general considerations before diving into details of the likelihoods used for inference.

First assume that the app's risk score r_e for an exposure window e represents a fair assessment of the risk of transmission during that window, up to an unknown constant κ that depends on factors unrelated to proximity/duration/timing, such as intrinsic transmissibility of the virus, susceptibility, immunity and vaccination status

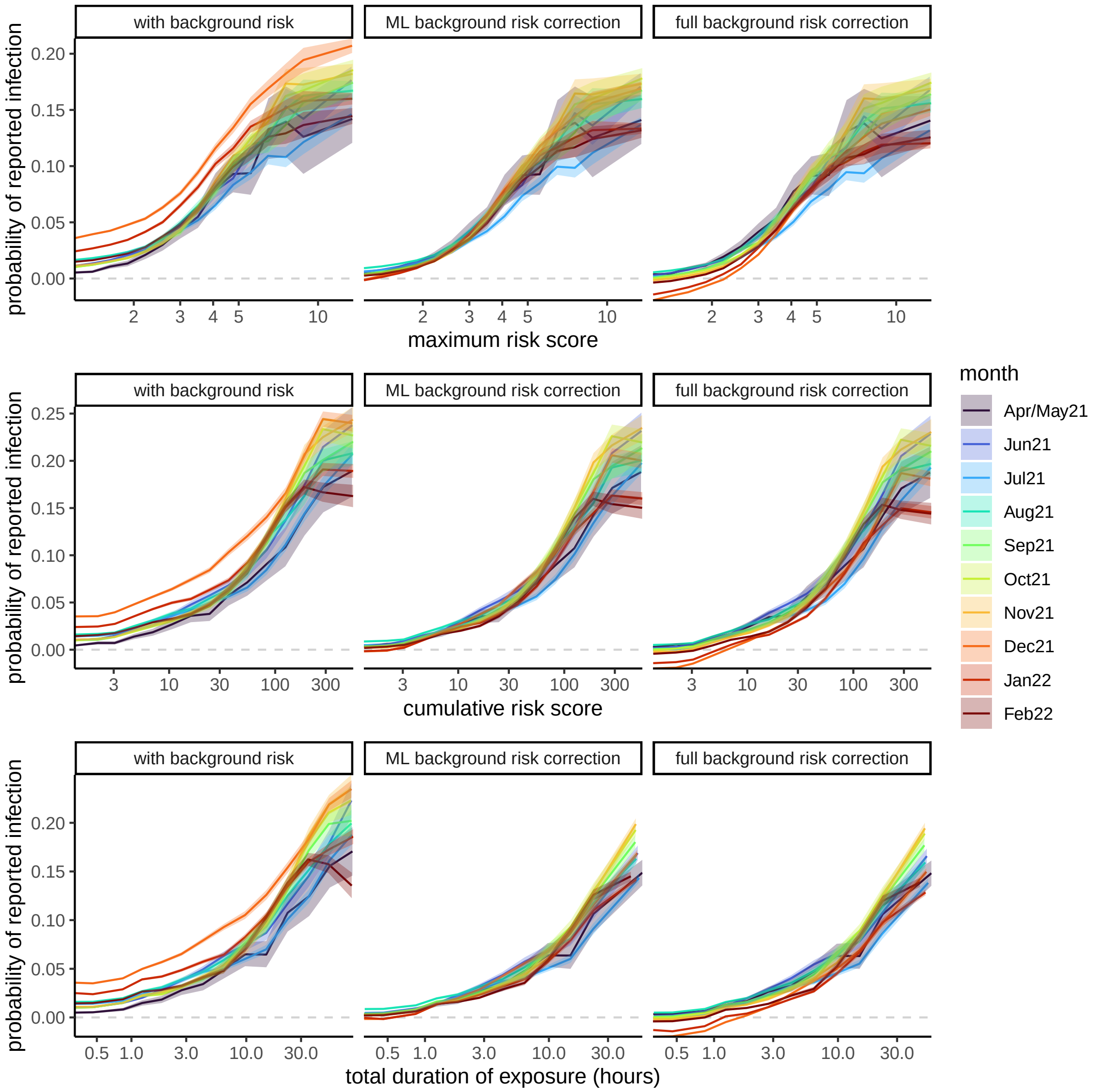

Figure S5: impact of partial and full background risk removal. The probability of reported infection (the fraction of notified contacts who report a positive test shortly after notification) is shown as a function of maximum risk score (top), cumulative risk score (middle) and duration (bottom), without correction (left), subtracting the maximum-likelihood correction for background risk (centre, corresponding to the probability of reported transmission), and subtracting the 'full' background risk, i.e. the naive incidence rate among all app users (right). The estimated full background risk is an overestimate, leading to some slightly negative risks in the right panel; for this reason, the maximum-likelihood correction is used throughout. Central values correspond to maximum likelihood estimates, shading indicates the 95% confidence intervals.

Pearson correlation coefficients	max risk score	mean risk score	duration	cumulative risk score	background risk	reported +ve test
max risk score	*1*	*0.76*	*0.59*	*0.69*	*0.02*	*0.15*
mean risk score	*0.76*	*1*	*0.23*	*0.37*	*0*	*0.08*
duration	*0.59*	*0.23*	*1*	*0.89*	*0.03*	*0.16*
cumulative risk score	*0.69*	*0.37*	*0.89*	*1*	*0.03*	*0.15*
background risk	*0.02*	*0*	*0.03*	*0.03*	*1*	*0.08*
reported +ve test	*0.15*	*0.08*	*0.16*	*0.15*	*0.08*	*1*

Table S3: Pearson correlation between summary statistics calculated per contact.

Spearman's correlation coefficients	max risk score	mean risk score	duration	cumulative risk score	background risk	reported +ve test
max risk score	*1*	*0.95*	*0.55*	*0.82*	*0.02*	*0.11*
mean risk score	*0.95*	*1*	*0.35*	*0.66*	*0.02*	*0.08*
duration	*0.55*	*0.35*	*1*	*0.87*	*0.02*	*0.11*
cumulative risk score	*0.82*	*0.66*	*0.87*	*1*	*0.03*	*0.12*
background risk	*0.02*	*0.02*	*0.02*	*0.03*	*1*	*0.07*
reported +ve test	*0.11*	*0.08*	*0.11*	*0.12*	*0.07*	*1*

Table S4: Spearman correlation between summary statistics calculated per contact.

of the contact, protection such as face masks, context of the exposure, etc. Under the simplest assumption, transmission can stochastically occur in any of the exposure windows. In this case, the probability of transmission to individual i is

$$P[\text{transmission to } i] = 1 - \prod_{e \in E_i} (1 - \kappa r_e) \tag{10}$$

For realistic modelling we should include arbitrary heterogeneities in the true risk that are not recorded by the app, both at the level of single exposure windows or individual contacts. There are also ascertainment biases in the reporting of positive test results. The most general model including both effects is:

$$P[\text{transmission to } i] = a_i \left[1 - \prod_{e \in E_i} (1 - H(\kappa r_e, h_{e,i})) \right] \tag{11}$$

with $H(0, h) = 0$ (i.e. true risk vanishes when the app risk score vanishes). The shape of the heterogeneity function $H(\cdot, h)$ in terms of the heterogeneity parameter h is implicitly defined by assuming that $H(x, h) \approx hx$, i.e. h parametrises the multiplicative heterogeneity for small risks of transmission. The unknown distribution for the heterogeneity between exposures windows for contact i, $h_{e,i}$, is denoted by $p_{\text{het},i}(h_{e,i})$, whose mean we denote by $h_i = \int_0^\infty dx \; p_{\text{het},i}(x)x$. The distribution of the per-contact mean heterogeneities h_i between contacts is denoted by $p_{\text{het}}(h_i)$, with mean $\bar{h} = \int_0^\infty dx \, p_{\text{het}}(x)x$, or $\bar{h}(F)$ if the mean is restricted to contacts with a specific feature F (such as having an exposure duration within a range of interest). We also denote the mean ascertainment factor among contacts by $\bar{a}$, or $\bar{a}(F)$ if the mean is restricted to contacts with a specific feature F.

Under the reasonable assumption that the typical risk during a single exposure window is very small $\kappa \bar{r}_e \ll 1$, we can approximate $1 - H(\kappa r_e, h_{e,i}) \approx 1 - h_{e,i} \kappa r_e \approx e^{-\kappa h_{e,i} r_e}$. Also assuming that the overall risk does not reach saturation (i.e $\kappa \bar{r}_e |E_i| < 1$), we obtain the probability of reported transmission

$$P[\text{transmission to } i] \approx a_i \left[1 - \exp\left(-\kappa \sum_{e \in E_i} h_{e,i} r_e \right) \right] \tag{12}$$

Averaging over the unrecorded heterogeneities between different exposures for the same individual and using the above approximation of small risk once more, we obtain

$$P[\text{transmission to } i] \approx a_i \left[1 - \exp\left(-\kappa h_i \sum_{e \in E_i} r_e \right) \right] = a_i \left[1 - e^{-\kappa h_i r_i^{\text{cum}}} \right] \tag{13}$$

i.e. the probability of reported transmission to i depends only on i's set of risk scores, $\{r_e : e \in E_i\}$, through the cumulative risk r_i^{cum}. Hence, on theoretical grounds, the cumulative risk score over all exposures of a contact should therefore be the best predictor of transmission to the contact.

Note however that both the individual heterogeneities h_i and ascertainment factors a_i can still be correlated to the same physical/behavioural factors entering the risk scores. For example, household contacts tend to have longer durations of exposure in closer proximity, but they also tend to engage in activities that are intrinsically more favourable to transmission of respiratory viruses, such as eating together or kissing, hence their risk of transmission is greater than what would be predicted by duration

and proximity alone. Also, contacts may be more or less likely to get tested when they are aware of another infection in their household, compared to the case when they are simply notified about an unknown exposure.

When we can ignore these correlations, the joint probability for a_i and h_i given r_i^{cum} is separable in a_i and h_i and independent of r_i^{cum}, i.e. $p(a_i, h_i | r_i^{\text{cum}}) = p(a_i)p(h_i)$. Using this to marginalise equation 13 over a_i and h_i, i.e. averaging over the unknown heterogeneities in proportion to their probability, we obtain

$$
\begin{aligned}
P[\text{transmission to } i] &\approx \int_{h_i}\int_{a_i} p(h_i)p(a_i)a_i \left[1 - e^{-\kappa h_i r_i^{\text{cum}}}\right] \, dh_i \, da_i &(14)\\
&\approx \left(\int_{a_i} p(a_i)a_i \, da_i\right)\left(1 - \int_{h_i} p(h_i)e^{-\kappa h_i r_i^{\text{cum}}} dh_i\right) &(15)\\
&\approx \bar{a}\left[1 - Z_{\text{het}}(\kappa r_i^{\text{cum}})\right] &(16)
\end{aligned}
$$

where $Z_{\text{het}}(y) = \int_0^\infty dh \; p_{\text{het}}(h)e^{-yh}$ is the Laplace transform of the distribution of heterogeneities across contacts (i.e. $Z_{\text{het}}(-y)$ is its moment-generating function). This result is the basis for the maximum-likelihood inference of the transmission risk per exposure window in this paper.

Saturation of the probability of reported transmission occurs for large cumulative risk, i.e. when $\kappa r^{\text{cum}}\bar{h} \gg 1$. In this regime, the probability does not grow linearly with r^{cum} but converges to a maximum value given by $\bar{a}$. Since $\bar{a} \sim 0.3 - 0.5$ for the UK during this period, the nonlinearities observed e.g. in Extended Data Figure 2 could be explained by saturation.

If the cumulative risk score is small, we can simplify the risk of transmission as

$$
P[\text{transmission to } i] \approx \kappa\bar{a}\bar{h}r_i^{\text{cum}} = \kappa\bar{a}\bar{h}|E_i|\bar{r}_i \approx \frac{\kappa\bar{a}\bar{h}}{\bar{d}_e} \cdot d_i \cdot \bar{r}_i \qquad (17)
$$

showing that for low secondary attack rates, the impact of duration d_i and mean risk score $\bar{r}_i$ on the true risk of transmission is approximately multiplicative.

If individual heterogeneities and ascertainment biases depended mostly on duration of exposure, we would still have a dependence of risk on duration and mean risk score that is multiplicative, albeit with a non-linear dependence on duration:

$$
P[\text{transmission to } i] \approx \frac{\kappa}{\bar{d}_e} \cdot \bar{a}(d_i)\bar{h}(d_i)d_i \cdot \bar{r}_i \qquad (18)
$$

In fact, the most likely bias in our data may be related to the different propensity to get tested for contacts exposed to known infected individuals. When individuals got notified, no further information about their risk or their exposures was provided, hence the notification itself could not cause this bias. Notifications may still have a global effect, e.g. increasing the propensity of contacts to get tested upon symptoms (or upon notification after August 2021, after which notified contacts were eligible for testing even without symptoms) and therefore reducing the under-ascertainment in reported test positivities. However, if risk factors such as duration also increase the chance of informal or manual contact tracing, and if informal/manual tracing increases in turn the propensity to get tested, this may have generated a correlation between ascertainment and risk, and therefore an ascertainment factor growing e.g. with duration of exposure $\bar{a}(d)$.

While we have no direct way to detect such correlations in our data, their impact on our results is likely to be limited. The reason is the following. The ascertainment

bias in the whole UK population - i.e. the fraction of cases among all SARS-CoV-2 infections - is 1 case for every 2-3 infections. This ascertainment bias is mostly related to the probability of taking a COVID-19 test after symptoms or knowledge of exposure. For app users, notifications or parallel forms of tracing are likely to reduce this bias. Biases in reporting positive tests should be irrelevant since they should be independent of the exposure. Hence differential ascertainment in testing among contacts (i.e. the variation in $\bar{a}(d)$) is unlikely to account for a factor of more than 2 in the apparent risk of infection. This should be contrasted with the observed variation in probability of infection, which spans two orders of magnitude.

1.6 Statistical modelling of the per-exposure-window probability of transmission

1.6.1 The main model

We developed a framework to estimate the probability of transmission during a single exposure window (followed by the individual testing positive and reporting this through the app during the observation window), denoted P_t. Our framework followed (Murphy et al., 2021) in assuming that transmission can happen independently during each exposure window, as detailed in Section 1.5. It accounts for the cumulative risk of transmission due to multiple exposures, as well as the background risk from unrecorded exposures or exposures to infected individuals who did not use the app (or did not use it correctly). We modelled P_t as depending only on the exposure window's risk score r.

We modelled the background risk as proportional to the daily rate of positive tests reported through the app per non-notified app user in the same LTLA and on the same day of notification as the individual considered. This rate is available from the daily analytics packets, which indicate whether the user reported a positive test through the app that day and whether they have been recently notified. We took the weekly moving mean daily rate of positive tests to obtain a less noisy estimate, then summed it over a fortnight after the notification date of the contact in question, n_i, to obtain the background risk estimate b_i. We modelled the probability of a notified user testing positive due to their background risk b_i as $B_i = 1 - (1 - b_i)^\beta$, parameterised with the constant $\beta > 0$ to be estimated. For small values of βb_i, the background risk is simply rescaled by a factor β, i.e. $B_i \approx \beta b_i$. However, for larger values, the functional form accounts for saturation of risk.

In the absence of ascertainment bias, the likelihood for individuals testing positive ($\pi_i = 1$) or not ($\pi_i = 0$) is

$$L = \prod_{i \in I} \left[(1 - B_i) \prod_{e \in E_i} (1 - P_t(r_e)) \right]^{1-\pi_i} \left[1 - (1 - B_i) \prod_{e \in E_i} (1 - P_t(r_e)) \right]^{\pi_i} \quad (19)$$

To minimise potential issues with fleeting exposures and with risk saturation (long exposures), we included only individuals with a few risky exposure windows (and correspondingly low risk of transmission) in the likelihood. Specifically, we required multiple risky exposures but no more than 3 hours of them, i.e. $2 \leq |E_i| \leq 6$. This also mitigates the issues caused by correlations between unrecorded heterogeneities in contacts and their duration/risk.

We estimated the function $P_t(r)$ and the parameter β (and also a and the heterogeneity parameters described in section 1.6.2 for those analyses) by likelihood

maximisation, starting the optimisation algorithm from 10 different random starting points around the typical probability of transmission and ensuring convergence until an accuracy of 0.1% on the final value is reached. The 95% frequentist confidence intervals (CIs) were estimated by likelihood profiling using likelihood ratio tests. We modelled the function $P_t(r)$ as piecewise constant, allowing discontinuous changes at r values of $4/3, 14/9, 16/9, 20/9, 30/9, 40/9, 50/9$.

Analogously to our calculation of a reference value for individual-level probability of testing positive, we calculated a reference value for the exposure-window-level probability of testing positive, $\hat{P}_t(r = 1)$, again using quadratic regression extrapolation on the results of the binned maximum-likelihood analysis in Figure 3 restricted to bins with $r < 3$.

For Figure 3, we imposed the condition that the relation between risk scores and risk was monotonically increasing, i.e. $P_t(r_1) \leq P_t(r_2)$ if $r_1 < r_2$. We used the same assumption to compute the maximum-likelihood individual background risks $B_i^{ML} = 1-(1-b_i)^{\beta_{ML}}$. We did not assume this condition to be true for any robustness analysis.

1.6.2 Incorporating ascertainment bias and risk heterogeneity

We checked the robustness of our results by extending the model to include more realistic risk structure, namely ascertainment bias in testing positive and heterogeneity in risk among individuals. This extended model is based on equation (16) and the underlying theory discussed in Section 1.5.

With an ascertainment parameter $a \in (0, 1]$, the likelihood for individuals testing positive ($\pi_i = 1$) or not ($\pi_i = 0$) is

$$L = \prod_{i \in I} \left[1 - a + a(1 - B_i) \prod_{e \in E_i} (1 - P_t(r_e)) \right]^{1-\pi_i} \times \left[a \left(1 - (1 - B_i) \prod_{e \in E_i} (1 - P_t(r_e)) \right) \right]^{\pi_i} \tag{20}$$

Whether an individual becomes infected or not, given our observations of their exposure, is effectively a stochastic outcome. However, given certain incomplete information about the exposure, such as app-recorded risk scores, variability in other unrecorded aspects of the exposure will causally affect the probability of the outcome and whether we can observe it via a positive test result. Some of these factors contributing to risk heterogeneity will be effectively constant for all of the recorded exposure windows for a given exposed individual i, i.e. will be the same for all exposure windows $e \in E_i$, for example immunity, a general tendency towards precautionary behaviour, and an inclination to test and report if positive. Other factors could vary between these windows, e.g. a long exposure event could consist of some windows indoors and some outdoors, some with face masks and some without, etc. Unmodelled heterogeneity in risk factors between different exposure windows for the same individual is effectively absorbed into greater stochasticity in individuals' outcomes. However, risk heterogeneity at the level of the individual can be modelled by scaling risk differently for different individuals with the same scaling h_i for all exposure windows $e \in E_i$ for individual i. Specifically, we modelled individual risk heterogeneity by modifying the per-exposure-window risk of testing positive for window e for individual i to be $P_t(r_e) = 1 - e^{-h_i \tau(r_e)}$. This functional form is consistent

with a multiplicative dependence on heterogeneity for small probabilities of transmission, and with saturation of risk when the probability is large. The function $\tau(r_e)$ is the estimand of interest, quantifying how per-window risk varies with the app's risk score, and the set of values $\{h\}$ over all our notified individuals $i \in I$ is a nuisance parameter to be integrated out. Let $p_{\text{het}}(h)$ be the distribution of values of h over individuals. We marginalised the nuisance parameters out of the likelihood, integrating out each h_i parameter and thus averaging each term $\prod_{e\in E_i}(1 - P_t(r_e))$ in the likelihood. Equation (20) becomes:

$$
\begin{aligned}
L = \prod_{i\in I} \int dh_i\, p_{\text{het}}(h_i) \left[1 - a + a(1 - B_i)e^{-h_i \sum_{e\in E_i} \tau(r_e)}\right]^{1-\pi_i} \times \\
\left[a\left(1 - (1 - B_i)e^{-h_i \sum_{e\in E_i} \tau(r_e)}\right)\right]^{\pi_i} \\
= \prod_{i\in I} \left[1 - a + a(1 - B_i) \int dh_i\, p_{\text{het}}(h_i)e^{-h_i \sum_{e\in E_i} \tau(r_e)}\right]^{1-\pi_i} \times \\
\left[a\left(1 - (1 - B_i) \int dh_i\, p_{\text{het}}(h_i)e^{-h_i \sum_{e\in E_i} \tau(r_e)}\right)\right]^{\pi_i} \qquad (21)
\end{aligned}
$$

where the simplification arises because each π_i is either 0 or 1.

We considered two distributions for the heterogeneity in individual risk p_{het}:

- a two-parameter mixture of individuals at maximum relative risk, at intermediate relative risk varying uniformly between 0 and 1, and at no risk:

  $$p_{\text{het}}(h) = (1 - p_0)p_m\delta(h - \bar{h}) + (1 - p_0)(1 - p_m)U_{[0,\bar{h}]}(h) + p_0\delta(h) \qquad (22)$$

  where $\bar{h}(1-p_0)(1+p_m)/2 = 1$ and $\delta(\ldots)$ is the Dirac delta function. This results in the integral in equation (21), i.e. the Laplace transform of equation (22), evaluating to

  $$
  \begin{aligned}
  \int dh_i\, p_{\text{het}}(h_i)e^{-h_i \sum_{e\in E_i} \tau(r_e)} = \\
  p_0 + (1 - p_0)p_m e^{-\sum_{e\in E_i} \tau(r_e)} + (1 - p_0)(1 - p_m)\frac{1 - e^{-\sum_{e\in E_i} \tau(r_e)}}{\sum_{e\in E_i} \tau(r_e)} \qquad (23)
  \end{aligned}
  $$

- a two-parameter mixture of individuals with Gamma-distributed risk and individuals not at risk:

  $$p_{\text{het}}(h) = (1 - p_0)\text{Gamma}(h|k, \theta) + p_0\delta(h) \qquad (24)$$

  with $k\theta(1 - p_0) = 1$. This results in the integral in equation (21) evaluating to

  $$\int dh_i\, p_{\text{het}}(h_i)e^{-h_i \sum_{e\in E_i} \tau(r_e)} = p_0 + \frac{1 - p_0}{\left(1 + \theta \sum_{e\in E_i} \tau(r_e)\right)^k} \qquad (25)$$

Maximum Likelihood parameter estimates and AIC values for these distributions are reported in Table S5.

In one analysis we also considered different mappings between risk score r and true risk $1 - e^{-\tau(r)}$ for different days of the week or different geographical areas (Figures S6 and S7). For that analysis, when a given contact was exposed over multiple days, we used the day of the week with most exposure windows as the day of the exposure. We grouped geographical areas depending on the rural/urban score of the local authority (ONS, 2011) : "rural" (score 1-2),"urban" (score 3-4) and "conurbation" (score 5-6).

Table S5: AIC values (with respect to the standard analysis) and Maximum Likelihood parameter estimates for several distributions of risk heterogeneity, with or without ascertainment bias. If the model includes ascertainment bias, we report its Maximum Likelihood estimate.

Distribution	Ascertainment?	AIC	parameters
No heterogeneity	no	ref	none
No heterogeneity	yes, $\hat{a} = 0.97$	1.8	none
Zero-inflated maximum+uniform	no	3.4	$\hat{p}_0 = 0, \hat{p}_m = 0.88$
Zero-inflated maximum+uniform	yes, $\hat{a} = 0.81$	-163.9	$\hat{p}_0 = 0.94, \hat{p}_m = 1$
Zero-inflated Gamma	no	-170.3	$\hat{p}_0 = 0.95, \hat{\theta} = 0.26$
Zero-inflated Gamma	yes, $\hat{a} = 1$	-168.3	$\hat{p}_0 = 0.95, \hat{\theta} = 0.26$

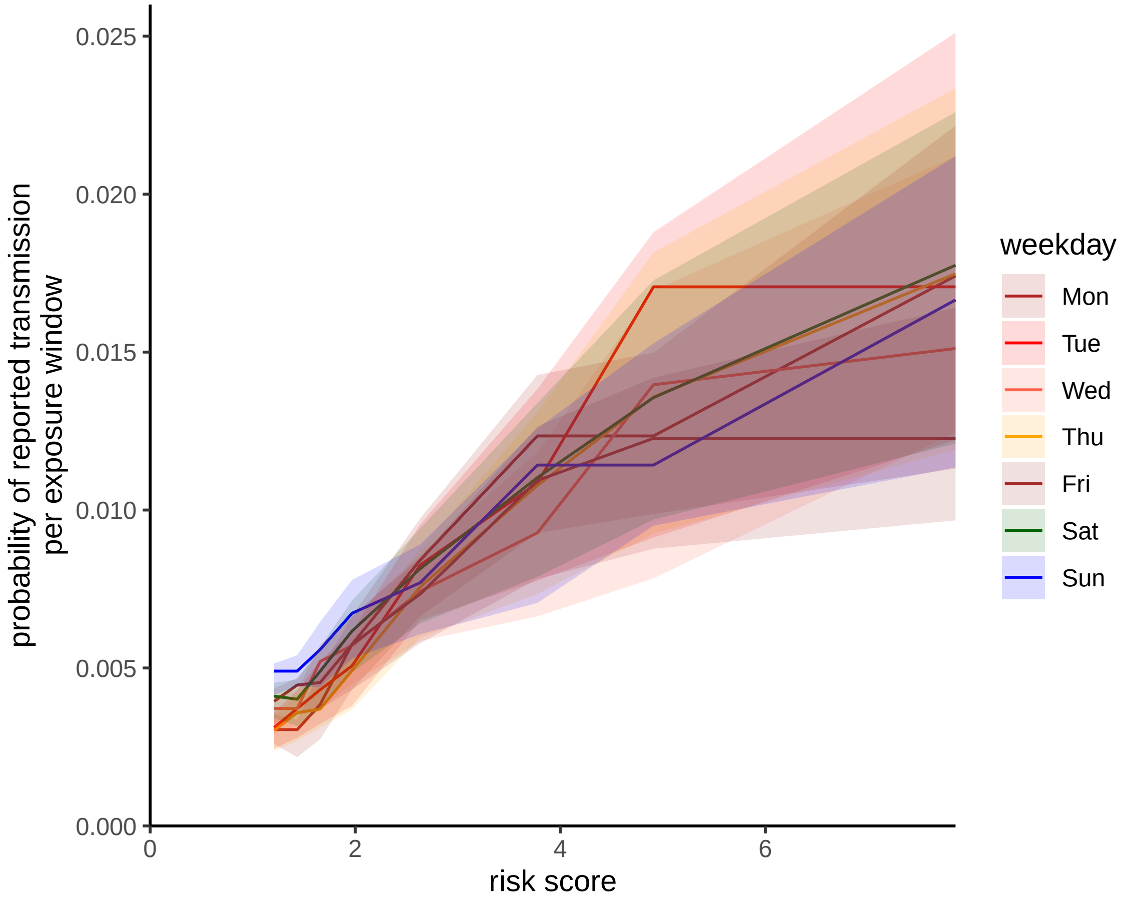

Figure S6: the same as Extended Data Figure 4a, but each day of the week is shown separately. Central lines represent maximum likelihood estimates, shading indicates 95% CIs.

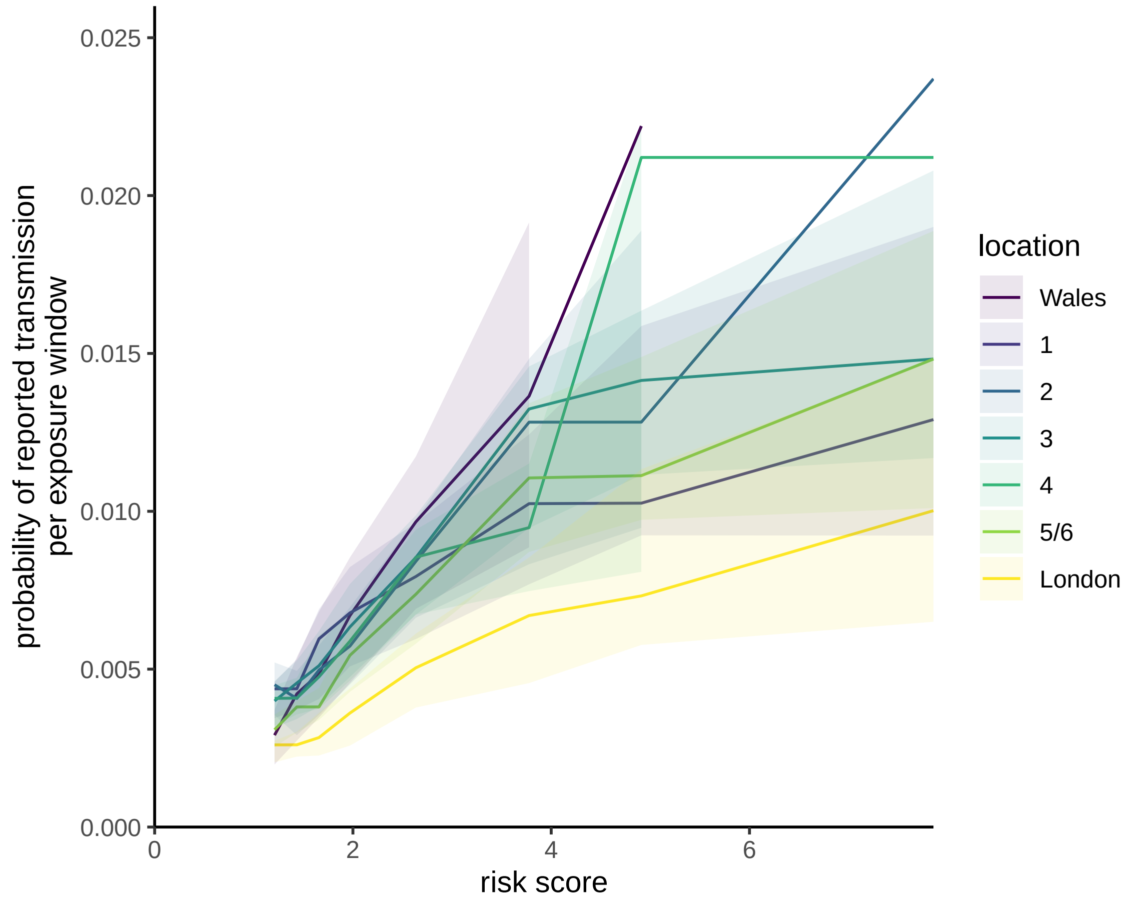

Figure S7: the same as Extended Data Figure 4b, but each rural/urban category is shown separately, and the result for exposures in Wales is also shown. The category “5/6” refers to all local authorities classified as “5” or “6” but not included in London, while the latter is shown separately. Central lines represent maximum likelihood estimates, shading indicates 95% CIs.

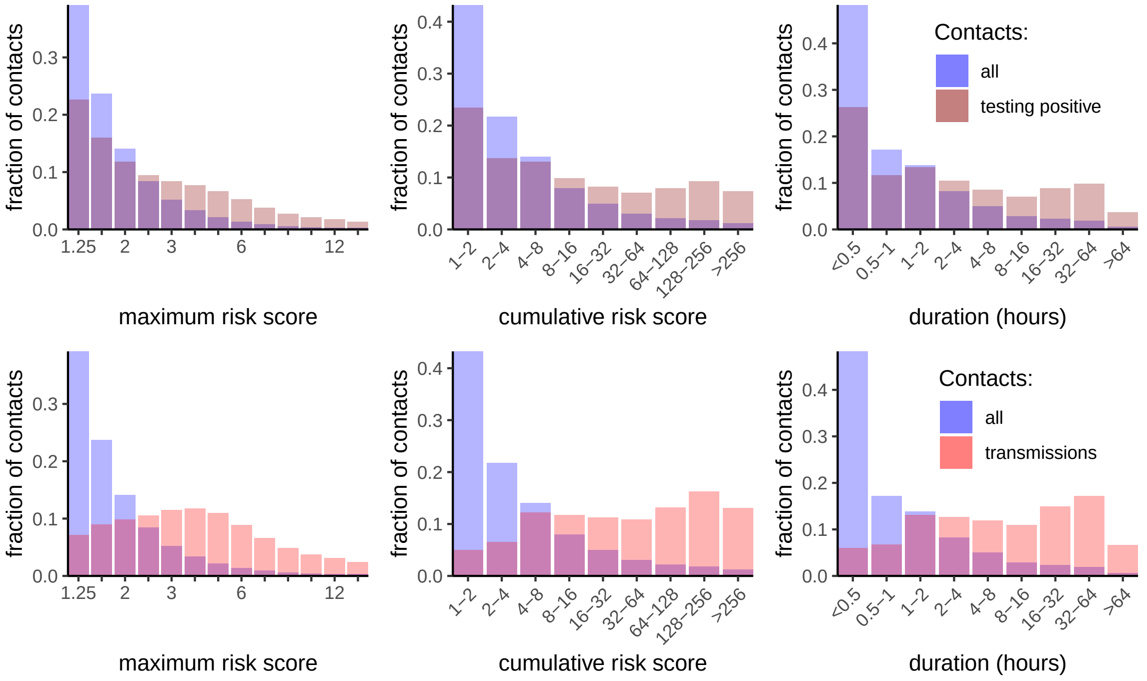

Figure S8: Distributions over all contacts (recalling that we analysed only *notified* contacts) of three summary metrics for their app-recorded exposure measurements, namely the maximum risk score for any of the exposure windows, the cumulative risk score over all exposure windows, and the total duration (summed over exposure windows). Distributions are shown separately for all contacts (blue), for only those contacts who reported a positive test result through the app in the observation window (brown, top row of plots), and for only those contacts who reported a positive test result through the app in the observation window and for whom we attributed the transmission to the recorded exposure rather than the background risk (red, bottom row of plots).

1.7 Distributions of predictors

The binned distribution for the three predictors (maximum risk score, duration, cumulative risk score) is shown in Figure S8. Kernel density estimates for log-maximum risk score were based on a Gaussian kernel. Kernel density estimates for cumulative risk score and duration in Figure 4 in the Main Text were obtained by first transforming the data through a piecewise linear-log transformation $f \in C^1$ defined as

$$f(x) = \begin{cases} x & x \leq 10 \\ 10(1 + \log(x/10)) & x > 10 \end{cases}$$

then using a shifted Gaussian kernel $N(0.5, 1)$ to correct in part for local right-censoring effects due to short windows (for example, an exposure of 40 minutes at 2 metres' distance would be recorded as a risky exposure window of 30 minutes, discarding the final 10 minutes). Kernel bandwidth was chosen as the minimum bandwidth for which the artificial local non-monotonicity due to discrete windows was not visible in the plots. Finally, densities were transformed back to a log scale with the appropriate Jacobian.

Distributions of risk predictors among "transmissions" were corrected for background risk. First, binned counts (or non-normalised densities) were computed for contacts reporting a positive test. Then, the mean contribution of background risk was computed by summing the maximum-likelihood background risk estimate $1-(1-$

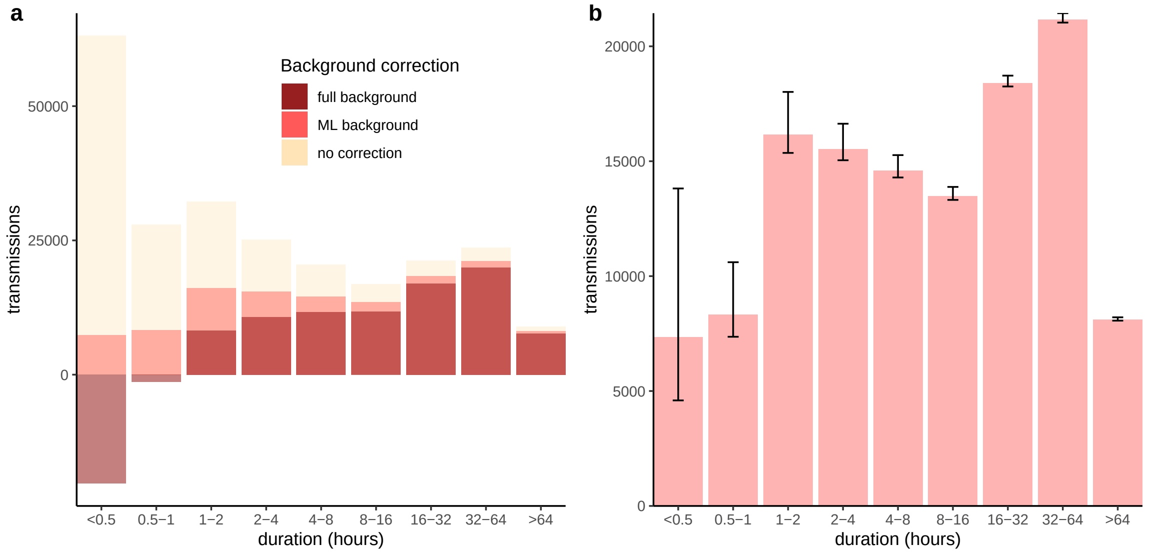

Figure S9: Distribution over contacts (recalling that we analysed only notified contacts) of the total duration of their exposure to an index case. Panel a: raw counts are shown as heights of the bars in yellow, raw counts minus the maximum-likelihood (ML) background risk are shown in red, and raw counts minus the estimated full background risk are shown in dark red. The estimated full background risk is an overestimate, leading to some slightly negative transmission counts; for this reason, the maximum-likelihood correction is used throughout. Subtracting the background risk means we can attribute the remaining transmissions to their recorded exposures. Panel b: the height of the bars represents the ML result as in panel a, with error bars indicating the 95% confidence interval on the background correction.

$b_i)^{\beta_{ML}}$ for all contacts in each bin. This contribution was then subtracted from the binned counts. For the case of duration of exposure, Figure S9a shows the impact of subtracting nothing or subtracting the full contribution b_i instead; Figure S9b shows the 95% CIs from the uncertainty on β_{ML}.

1.8 Predicting which contacts report positive tests

Exposure notifications were sent by the app to an app-user if they had at least one exposure window that (a) connects them to another user who reported a positive test through the app and consented to share keys, and (b) has a risk score above the threshold (which was 10/9 throughout our period of study). This is equivalent to using the maximum risk score among all of an individual's exposure windows as a predictor of reporting a positive test.

We investigated different predictors of testing positive, combining information from different exposure windows. We were only able to test their performance among individuals who were notified by the app, and only use data from exposure windows with risk scores over the threshold, since this is all that was recorded in the dataset. We considered eight classifiers: the three summary statistics (maximum risk score $r_i^{\max}$, cumulative risk score r_i^{cum} and duration d_i) defined in equations 5-8, and three machine learning approaches (including three variants of XGBoost) for binary probabilistic classification:

- logistic regression

- Gradient Boosting Machine: classical approach implemented in the R library `gbm` (Greenwell et al., 2020)
- XGBoost (10, 100 and 400 rounds): gradient boosting approach implemented in the R library `xgboost` (Chen & Guestrin, 2016)

The predictors used as input for all machine learning classifiers were the counts of exposure windows in each of 8 risk score bins (10/9 - 4/3, 4/3 - 14/9, 14/9 - 16/9, 16/9 - 20/9, 20/9 - 30/9, 30/9 - 40/9, 40/9 - 50/9, 50/9 - ∞) and the summary statistics defined above in equations 5-8. The predicted (target) outcome is test positivity π_i.

An extra set of features with predictive potential investigated using the machine-learning classifiers was composed by

- date of notification n_i
- background risk b_i, i.e. background infection rate among app users in the same period and area
- peak daily duration of exposure, i.e. maximum number of exposure windows in the same day
- day of the week corresponding to the peak daily exposure i.e. to $\text{mode}_{e \in E_i}[t_e]$
- region of England, or Wales
- rural/urban score for each local authority in the scale 0-7, where 1-6 corresponds to the standard rural/urban classification at the local authority level for England (ONS, 2011) plus additional values of 0 for local authorities in Wales and 7 for local authorities in London.

We used a random subset of 2,000,000 contacts for machine learning. Exposure data from half of these individuals were used for training, the other half for validation.

2 Supplementary Discussion

2.1 Predictive power of app-measured scores for classification of contacts

We explored the effects of using different criteria for the binary risk classification of who is notified and who is not. The NHS app classified contacts as requiring notification or not based on its maximum recorded risk score in any of the separately analysed 30-minute exposure windows. Criteria less strict than the one actually used—a maximum risk score of at least 1.11—could not be tested because our data was only for those individuals who were actually notified (data for unnotified individuals cannot be obtained retrospectively). Criteria more strict than the one actually used were tested by hypothetically excluding subsets of individuals from notification, based on their measurements, and then examining the fraction of positives and not-positives who would have been notified among those not excluded. We tested the use of several metrics other than maximum risk score, as well as machine-learning classifiers based on the same metrics and on binned counts of risk scores, adding also extra information on background risk, date, geographical region, rural/urban classification of the local area, day of the week and duration of peak daily exposure. There was room for

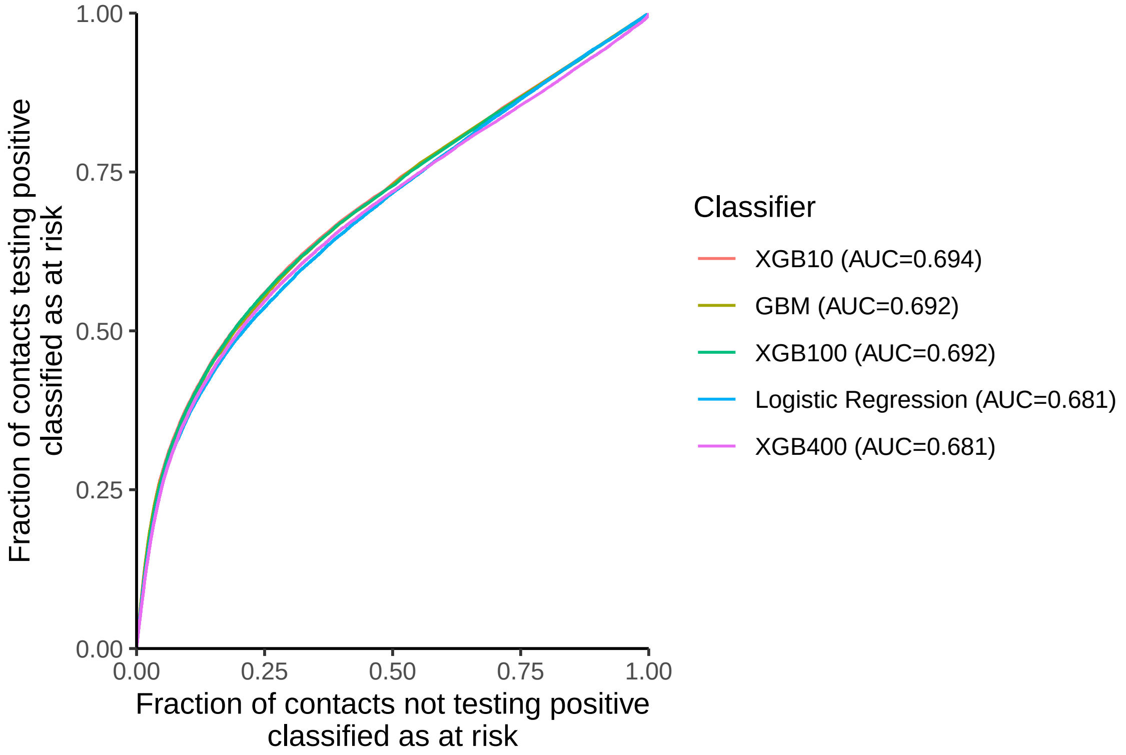

Figure S10: Receiver operating characteristic curve for different methods and thresholds to classify individuals exposed to an index case into notified or unnotified. Our dataset contained only individuals who were actually notified; we varied the classification thresholds to interpolate between continuing to notify all of these individuals (top right) and notifying none of these individuals (bottom left). Different colours show different machine-learning classifiers, for each of which we varied thresholds to explore their possible balance between sensitivity (notifying individuals who would report a subsequent positive test) and specificity (not notifying individuals who would not). AUC in the label abbreviates area under the curve (a metric of classification success).

a small improvement in classification by using cumulative risk or duration instead of maximum risk. Either of these performed nearly as well as the best machine-learning method (Extended Data Figure 5). All machine-learning methods tested had similar performance (Figure S10), and the only significant further gain from additional information came from the inclusion of background risk (Figures S11,S12). In fact, duration and background risk alone were enough for a near-optimal prediction with an area under the curve of 0.73; the addition of all the other predictors increased it only very slightly to 0.74.

Getting the balance between sensitivity and specificity right is generally difficult in infection control (J. Petrie and Masel, 2021) because of non-linear epidemic dynamics: preventing more transmissions in the short term may prevent many more notifications further down the transmission chain.

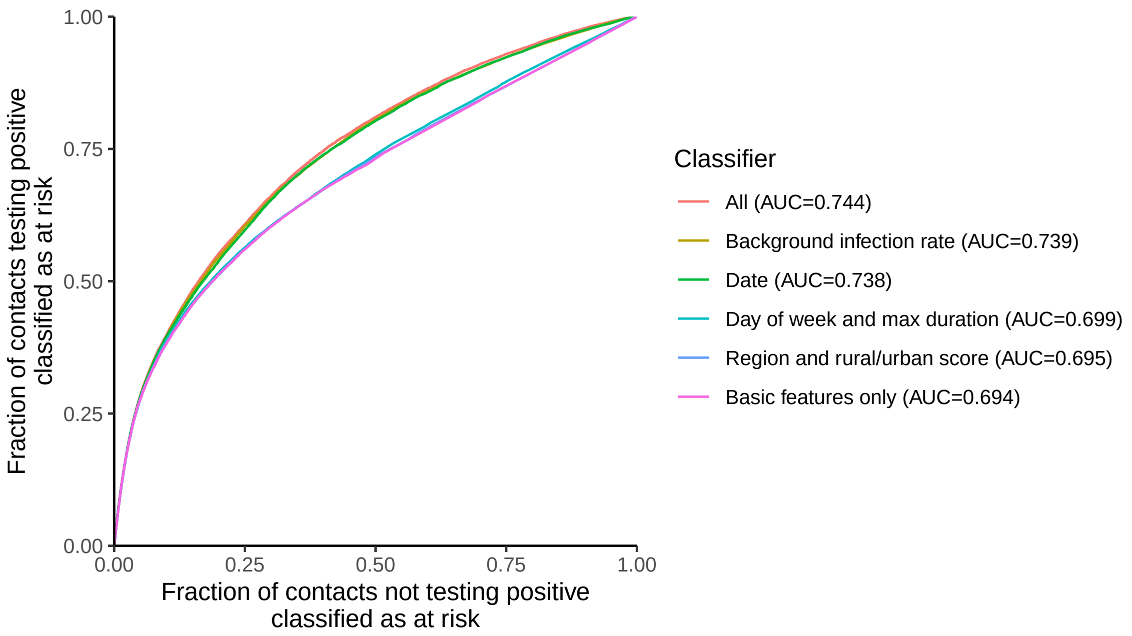

Figure S11: How the receiver operating characteristic curve for the best machine-learning classifier (XGB10) changes by adding one extra feature (indicated in the legend) to the basic predictors (background risk, date, day of the week for peak exposures and maximum daily duration of exposure, region of England and rural/urban classification score of the local authority). We also show the curve obtained from all predictors and from basic predictors only.

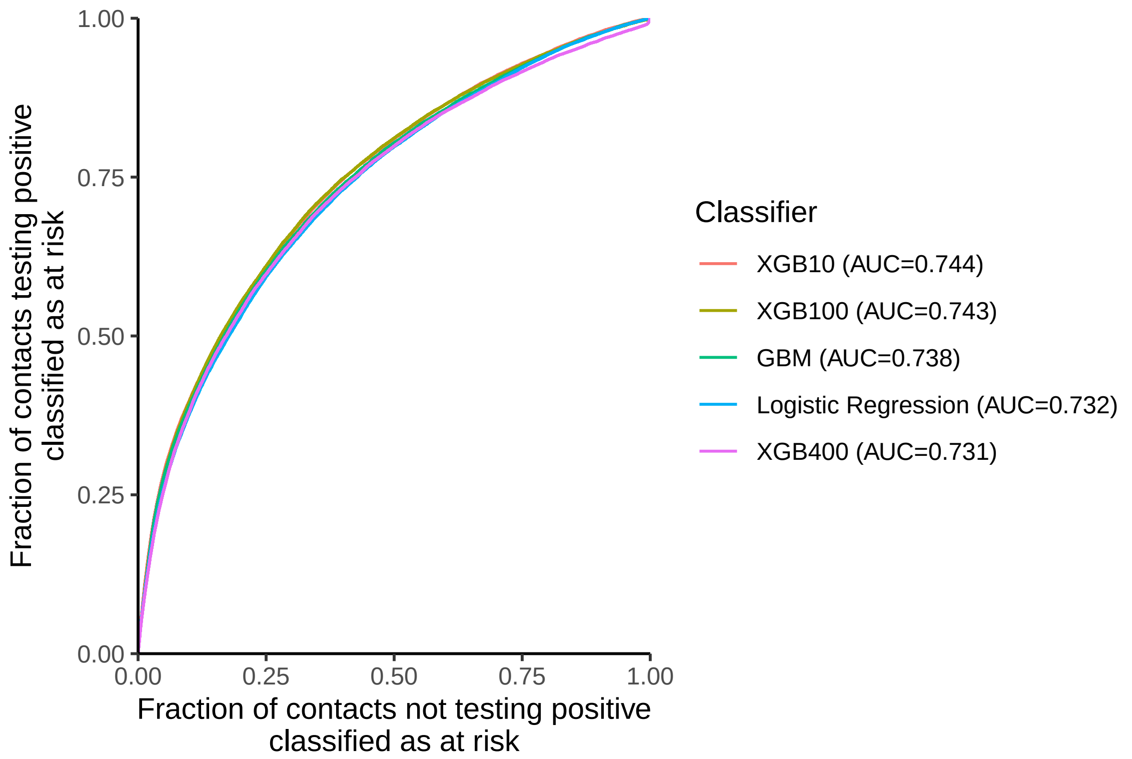

Figure S12: the same as Figure S10, but adding extra features to the predictors: background risk, date, day of the week for peak exposures, maximum daily duration of exposure, region of England and rural/urban classification score of the local authority.

2.2 Optimising interventions for contacts with intermediate risk of infection

Using information on the risk of infection given duration of exposure, it is possible to evaluate the impact of alternative contact tracing policies for COVID-19. In Section 2.2.1 we show that by adding the option for recommendation of testing instead of quarantine ("amber notification"), the cost of preventing transmissions can be reduced. In Section 2.2.2 we show that the optimal strategy for delivering post-exposure prophylaxis to contacts depends on the duration of exposure and the risk of hospitalisation conditional on infection.

An extended version of this framework for optimisation of epidemic measures can be found in J. I. M. Petrie and Masel, 2023.

2.2.1 Amber notification and testing of contacts

As discussed in the Main Text, duration of close exposure is the main predictor of transmission, and it is easy to determine from either digital contact tracing or from the individual's recall (as in manual contact tracing). At the beginning of the COVID-19 pandemic, most countries opted for a contact tracing strategy with just two options: no interventions for low-risk contacts, or quarantine for high-risk ones. Within this strategy, the best choice would be to quarantine all contacts with a duration of exposure above a given threshold. This baseline threshold on duration d_b would be based on public health considerations and tradeoffs that we do not explore here.

Consider the scenario shown in Extended Data Figure 6, where instead of using a single threshold to decide between quarantine or no notification at all, there is an option for an intermediate level of risk at which testing is recommended without quarantine ("amber notification"). If the test result is positive, the contact becomes a case and should self-isolate.

The effectiveness of a recommendation depends on how much transmission it prevents. Define E_q as the expected number of transmissions prevented by quarantine (relative to doing nothing) per infected individual and E_t as the expected number of transmissions prevented by testing and only isolating after receiving a positive result.

We consider a scenario where the duration of exposure to an index case is determined for each contact and policies can target contacts based on that duration. A policy can be described by the durations d_t and d_q needed to recommend testing and quarantine respectively. The recommendation for a contact of duration D is then described by equation (26):

$$\text{Recommendation}(D) = \begin{cases} \text{Not traced,} & \text{if } D < d_t \\ \text{Test,} & \text{if } d_t \leq D < d_q \\ \text{Quarantine,} & \text{if } D \geq d_q \end{cases} \tag{26}$$

The effectiveness E and cost C of a policy then depend on the fraction of total and infected contacts that are assigned to each category. Equation (27) computes the effectiveness of a given policy as the expected reduction in transmissions per contact, while equation (28) computes the cost of a given policy based on the expected cost per contact:

$$E(d_t, d_q) = E_t \cdot \int_{d_t}^{d_q} p(D) \cdot P(\text{infected}|D) \cdot dD + E_q \cdot \int_{d_q}^{\infty} p(D) \cdot P(\text{infected}|D) \cdot dD$$

$$(27)$$

$$C(d_t, d_q) = C_t \cdot \int_{d_t}^{d_q} p(D) \cdot dD + C_q \cdot \int_{d_t}^{d_q} p(D) \cdot P(\text{infected}|D) \cdot dD + C_q \cdot \int_{d_q}^{\infty} p(D) \cdot dD \tag{28}$$

where $p(D)$ is the distribution of duration of exposure among contacts. Note that one of the terms in equation (28) depends on the probability of infection because those that test positive must isolate, which we assume has an equivalent cost to quarantine (i.e. the amount of quarantine required for someone receiving a positive test result some time after being traced (due to a testing delay) is the same as someone quarantining immediately upon being traced).

The choice of parameters d_t and d_q can then be optimized to either improve the effectiveness (equation 29) or reduce the cost (equation 30) compared to a default quarantine-only policy. For a baseline policy recommending quarantine at duration d_b with no testing option, the cost is $C_b = C(d_t = d_b, d_q = d_b)$ and the effectiveness is $E_b = E(d_t = d_b, d_q = d_b)$ [1]. The optimisation problems are therefore

$$\max_{d_q \in [0,\infty), d_t \in [d_q,\infty)} E(d_t, d_q) \quad \text{s.t. } C(d_t, d_q) \leq C_b \tag{29}$$

$$\min_{d_q \in [0,\infty), d_t \in [d_q,\infty)} C(d_t, d_q) \quad \text{s.t. } E(d_t, d_q) \geq E_b \tag{30}$$

Assume for simplicity $E_t/E_q = 0.5$; that is, testing without quarantine (followed by self-isolation if positive) prevents half as many transmissions as quarantine. The reduction in effectiveness could be due either to false negatives or to delays in getting results. The cost of the recommendation depends on the burden imposed by it. Assume $C_t/C_q = 0.1$, i.e. the cost of a test (including the financial cost and the time required to collect and take the test) for someone who is not infected is 10% of the cost of quarantine.

The resulting policy when we optimise the effectiveness of the intervention without increasing the baseline cost is shown in Extended Data Figure 7a, while the minimum cost policy that minimises overall costs without reducing baseline effectiveness is shown in Extended Data Figure 6a. Both approaches trace the same Pareto frontier, but at different reference points depending on the priority of the policy optimization. Extended Data Figure 7b demonstrates that by adding the option for this intermediate recommendation, the effectiveness can be increased by up to 60%. Similarly, Extended Data Figure 6b shows that the cost can be reduced by up to 40%.

2.2.2 Optimal strategy for post-exposure prophylaxis

Even when non-pharmaceutical interventions have been removed, risk assessment for contacts could still be relevant to prioritise treatment, for example for vulnerable individuals who are at high risk of hospitalisation if infected.

Post-exposure prophylaxis (PEP) is the use of drugs to prevent illness after potential exposure to a pathogen. There are several ongoing clinical trials for COVID-19

[1]Note that by setting the testing and quarantine threshold to the same value, no amber notifications are sent and no contacts are recommended to test.

PEP drugs, though no drug is currently authorised by the US National Institutes of Health. A 2022 trial by Pfizer found reductions of 32-37% in the risk of confirmed symptomatic COVID-19 infections, but results were not statistically significant (Pfizer, 2022). PEP could be a very useful resource for vulnerable individuals at very high risk of hospitalisation because of risk factors such as age and pre-existing health issues.

Since PEP drugs have a cost, it is worthwhile to evaluate when post-exposure prophylaxis is beneficial. We will show that whether it is beneficial strongly depends on the estimated risk of infection and the probability of severe disease conditional on infection. As discussed in the previous section, PCR or rapid testing can be used to gain more information about the probability that the individual at risk is actually infected.

If post-exposure prophylaxis were to be linked to contact tracing, our results could immediately suggest the most effective course of action depending on duration of exposure and risk of hospitalisation of the contact (Figure S12).

We consider a policy that would recommend either immediate PEP treatment or testing to vulnerable contacts, depending on their duration of exposures to a case; a positive test result would then trigger PEP. This policy corresponds to the following recommendation:

$$\text{Recommendation}(D|P_h) = \begin{cases} \text{Wait} & \text{if } D < d_t(P_h) \\ \text{Test to Treat with PEP} & \text{if } d_t(P_h) \leq D < d_p(P_h) \\ \text{Immediate PEP} & \text{if } D \geq d_p(P_h) \end{cases} \tag{31}$$

where d_t is the minimum duration of exposure for testing and d_p is the minimum duration of exposure for PEP. Both values depend on P_h, the probability of hospitalization conditional on infection. The optimal choice of d_t and d_p can be framed as an expected value optimization problem. The decision depends on the following additional parameters:

- C_h: The cost of hospitalization for severe disease
- C_p: The cost of PEP
- C_t: The cost of a test
- E_p: The effectiveness of PEP, measured as the relative reduction in hospitalizations conditional on infection (e.g. a drug with $E_p = 1$ would prevent all hospitalisations)
- P_t: The sensitivity of the test, i.e. the probability of a test result being positive conditional on the person being infected.

The expected cost of a given policy for a given P_h is given by equation (32):

$$\begin{aligned} C(d_t, d_p) &= C_h \cdot P_h \cdot \int_0^{d_t} p(D) \cdot P(\text{infected}|D) dD + C_t \cdot \int_{d_t}^{d_p} p(D) dD + \\ &+ P_t \cdot (C_p + (1 - E_p) \cdot C_h \cdot P_h) \int_{d_t}^{d_p} p(D) P(\text{infected}|D) dD + \\ &+ (1 - P_t) \cdot C_h \cdot P_h \int_{d_t}^{d_p} p(D) P(\text{infected}|D) dD + \\ &+ C_p \cdot \int_{d_p}^{\infty} p(D) dD + (1 - E_p) C_h \cdot P_h \int_{d_p}^{\infty} p(D) P(\text{infected}|D) dD \end{aligned} \tag{32}$$

There are several contributions to the cost to Equation (32): the cost of hospitalization for those advised to wait, the cost of testing, the cost of treatment for those that test positive, the (reduced) average cost of hospitalization for those that test positive, the original cost of hospitalization for those that test negative despite being infected, the cost of treatment for those who are given PEP, and the (reduced) average cost of hospitalization for those who are given PEP.

For a given value of P_h (which we can estimate based on information about the disease and the person's age and comorbidities), we can find the optimal thresholds d_t and d_p by solving the problem in equation (33).

$$\min_{d_t \in [0,\infty), d_p \in [d_t,\infty)} C(d_t, d_p) \tag{33}$$

Figure S13a shows the solution to this problem when $C_h = 60000$, $C_p = 600$, $C_t = 10$, $E_p = 0.5$ and $P_t = 0.5$. The figure shows the two duration thresholds depending on risk of hospitalization. At very low risk of hospitalization, it is optimal to wait even if infection is known. This is generally the case for a risk of hospitalisation lower than $P_h < C_{PEP}/(C_h \cdot E_{PEP})$. At intermediate risk of hospitalization, testing is recommended for most or all contacts. At very high risk of hospitalization, PEP without testing is recommended for contacts with a high risk of infection. Figure S13b shows that with these costs and effectiveness, the cost of the optimal policy is up to 30% lower than the wait-and-see policy.

References

ONS. (2011). *2011 rural/urban classification.* https://www.ons.gov.uk/methodology/geography/geographicalproducts/ruralurbanclassifications/2011ruralurbanclassification

Chen, T., & Guestrin, C. (2016). XGBoost: A scalable tree boosting system. *Proceedings of the 22nd ACM SIGKDD International Conference on Knowledge Discovery and Data Mining*, 785–794.

Briers, M., Charalambides, M., & Holmes, C. (2020). Risk scoring calculation for the current NHSx contact tracing app. http://arxiv.org/abs/2005.11057

Ferretti, L., Ledda, A., Wymant, C., Zhao, L., Ledda, V., Abeler-Dorner, L., et al. (2020). The timing of COVID-19 transmission. *MedRxiv, Publisher: Cold Spring Harbor Laboratory Press.* https://www.medrxiv.org/content/10.1101/2020.09.04.20188516v2

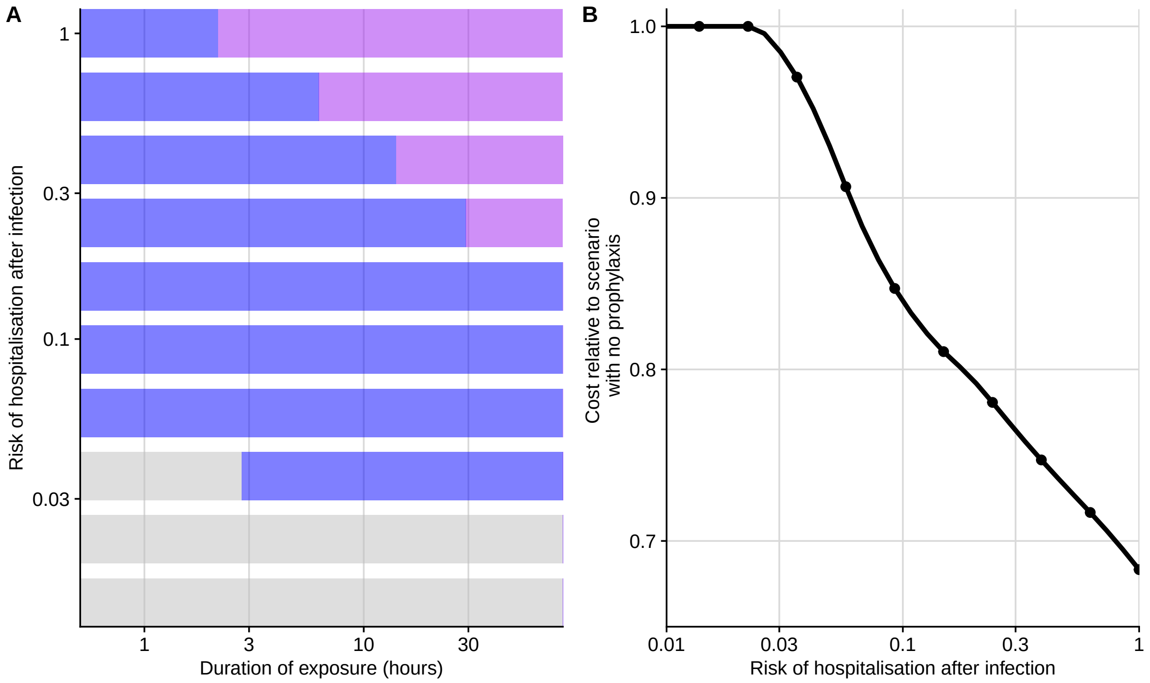

Figure S13: Optimal post-exposure treatment strategies depending on duration of exposure and degree of vulnerability of contacts. For illustrative purposes, we consider three strategies here: providing post-exposure prophylaxis (when available), testing the contact (providing treatment only if the test is positive), and doing nothing. Optimal strategies minimise the total cost for patient and public health. We assume that the risk of infection would be assessed based on duration of exposure. Panel a: each vertical line is a graphical representation of the optimal strategy for different classes of patients with a given baseline risk of hospitalisation if infected (x axis). Panel b: the reduction in costs of the optimal strategy relative to no intervention.

Fraser, C., Ferretti, L., Bonsall, D., Hinch, R., & Finkelstein, A. (2020). *Defining an epidemiologically meaningful contact from phone proximity events: Uses for digital contact tracing* (tech. rep.). https://github.com/BDI-pathogens/covid-19_instant_tracing/blob/master/Epidemiologically%20meaningful%20contact%20from%20phone%20proximity%20events%20-%20uses%20for%20digital%20contact%20tracing.pdf

Greenwell, B., Boehmke, B., Cunningham, J., & Developers, G. B. M. (2020). Gbm: Generalized boosted regression models.

Lovett, T., Briers, M., Charalambides, M., Jersakova, R., Lomax, J., & Holmes, C. (2020). Inferring proximity from bluetooth low energy RSSI with unscented kalman smoothers. http://arxiv.org/abs/2007.05057

Murphy, K., Kumar, A., & Serghiou, S. (2021). Risk score learning for COVID-19 contact tracing apps. In K. Jung, S. Yeung, M. Sendak, M. Sjoding, & R. Ranganath (Eds.), *Proceedings of the 6th machine learning for healthcare conference* (pp. 373–390). PMLR.

Petrie, J., & Masel, J. (2021). The economic value of quarantine is higher at lower case prevalence, with quarantine justified at lower risk of infection. *Journal of The Royal Society Interface*, *18*(182), 20210459.

Wymant, C., Ferretti, L., Tsallis, D., Charalambides, M., Abeler-Dörner, L., Bonsall, D., Hinch, R., Kendall, M., Milsom, L., Ayres, M., Holmes, C., Briers, M.,

& Fraser, C. (2021). The epidemiological impact of the NHS COVID-19 app. *Nature*, *594* (7863), 408–412.

Pfizer. (2022). *Press release: Pfizer shares top-line results from phase 2/3 epic-pep study of paxlovid for post-exposure prophylactic use.* https://www.pfizer.com/news/press-release/press-release-detail/pfizer-shares-top-line-results-phase-23-epic-pep-study

Kendall, M., Tsallis, D., Wymant, C., Di Francia, A., Balogun, Y., Didelot, X., Ferretti, L., & Fraser, C. (2023). Epidemiological impacts of the nhs covid-19 app in england and wales throughout its first year. *Nature Communications*, *14* (1), 858. https://doi.org/10.1038/s41467-023-36495-z

Petrie, J. I. M., & Masel, J. (2023). Optimal targeting of interventions uses estimated risk of infectiousness to control a pandemic with minimal collateral damage. *medRxiv*, 2023–10.

3 Pseudocode for processing raw app data

This R code illustrates unambiguously the set of processing steps we applied to the raw data to arrive at the form of the dataset analysed here.

```
library(tidyverse)

# In this illustration, data irrelevant for the study has already
# been discarded and variables have been renamed as follows.
#
# df_analytics is a dataframe containing one row for each app user
# and for each day, with the following columns:
# date_received (the date the data was was received)
# notified_today
# ltla
# postcode_district
# os_version (OS = operating sytem of the device)
# device_model
#
# df_events is a dataframe containing one row for each exposure window
# sent to the central server.
# There is one of these for each 30-minute window of risky exposure
# between a contact and an index case; e.g. a contact with 90 minutes
# of risky exposure to an index case will send three events packets
# (three rows in df_events) when they are notified.
# df_events has the following columns:
# ltla
# postcode_district
# os_version
# device_model (i.e. the same four individual-level characteristics
# as in df_analytics)
# risk_score
# BLE_details
# infectiousness_of_index
# duration (up to 30 minutes)
```

```
# date_exposure
# date (the date the packet was received, which should be the date of
# notification for notification-stage packets and the date of reporting
# of a positive test for test-positive-stage packets).
# positive_stage: when a contact reports a positive test during
# the observation window, they resend the same set of events packets
# as previously except with the logical column positive_stage
# now equalling TRUE instead of FALSE.

# Some toy data as an example
df_analytics <- tribble(
  ~date_received, ~ltla, ~postcode_district, ~os_version, ~device_model,
  "2021-01-01", "Oxford", "OX1", "iOS 1", "iPhone 10", FALSE, # user A
  "2021-01-02", "Oxford", "OX1", "iOS 1", "iPhone 10", TRUE, # user A
  "2021-01-03", "Oxford", "OX1", "iOS 1", "iPhone 10", FALSE, # user A
  "2021-02-01", "Oxford", "OX2", "android 1", "Galaxy S8", TRUE, # user B
  "2021-03-01", "Oxford", "OX3", "android 2", "Galaxy S6", TRUE, # user C
  "2022-01-01", "Cambridge", "CB1", "iOS 2", "iPhone 12", TRUE, # user D
  "2022-01-01", "Cambridge", "CB1", "iOS 2", "iPhone 12", TRUE # user E
)

df_events <- tribble(
  ~date_received, ~ltla, ~postcode_district, ~os_version, ~device_model,
  "2021-01-02", "Oxford", "OX1", "iOS 1", "iPhone 10", "details 1",
"high", 26, "2022-12-27", FALSE, # user A
  "2021-01-02", "Oxford", "OX1", "iOS 1", "iPhone 10", "details 2",
"high", 27, "2022-12-27", FALSE, # user A
  "2021-01-02", "Oxford", "OX1", "iOS 1", "iPhone 10", "details 3",
  "high", 28, "2022-12-27", FALSE, # user A
  "2021-01-10", "Oxford", "OX1", "iOS 1", "iPhone 10", "details 1",
"high", 26, "2022-12-27", TRUE, # user A
  "2021-01-10", "Oxford", "OX1", "iOS 1", "iPhone 10", "details 2",
"high", 27, "2022-12-27", TRUE, # user A
  "2021-02-01", "Oxford", "OX2", "android 1", "Galaxy S8", "details 4",
  "low",  25, "2021-01-29", FALSE, # user B
  "2021-03-01", "Oxford", "OX3", "android 2", "Galaxy S6", "details 5",
"low",  30, "2021-02-28", FALSE, # user C
  "2021-03-02", "Oxford", "OX3", "android 2", "Galaxy S6", "details 5",
"low",  30, "2021-02-28", TRUE, # user C
  "2022-01-01", "Cambridge", "CB1", "iOS 2", "iPhone 12", "details 6",
  "high", 22, "2021-12-28", FALSE, # user D
  "2022-01-01", "Cambridge", "CB1", "iOS 2", "iPhone 12", "details 7",
  "low", 27, "2021-12-26", FALSE # user E
)

# Identify the subset of app users and days when that app user was
# notified that day.
# The %>% operator passes the preceding object through the steps that
# follow.
df_analytics_notified = df_analytics %>%
```

```
  filter(notified_today == TRUE) %>% # Keep only rows with this condition
  select(-notified_today) %>% # remove the now-unwanted column
  rename(date_notified = date_received)

# Identify the subset of notifications when only a single contact
# notified on a given day has a particular combination of
# individual-level characteristics
df_analytics_notified_unique = df_analytics_notified %>%
  filter(.by = c(date_notified, ltla, postcode_district, os_version,
                 device_model),
         n() == 1)

# Separate events packets into those sent at the notification stage
# and those sent at the test-positive stage
df_events_notification = df_events %>%
  filter(positive_stage == FALSE) %>%
  select(-positive_stage) %>%
  rename(date_notified = date_received)
df_events_test_positive = df_events %>%
  filter(positive_stage == TRUE) %>%
  select(-positive_stage)  %>%
  rename(date_positive = date_received)

# For each notification-stage events packet, define a variable
# 'positive_packet' to indicate whether or not that exact same packet
# was re-sent at the test-positive stage, and if so, record when that
# was (date_positive).
df_events_notification_resent = inner_join(
  df_events_notification,
  df_events_test_positive,
  by = join_by(ltla, postcode_district, os_version, device_model,
         BLE_details, infectiousness_of_index, duration, date_exposure)
  ) %>%
  add_column(positive_packet = TRUE)
df_events_notification_not_resent = anti_join(
  df_events_notification,
  df_events_test_positive,
  by = join_by(ltla, postcode_district, os_version, device_model,
         BLE_details, infectiousness_of_index, duration, date_exposure)
  ) %>%
  add_column(positive_packet = FALSE)
df_events_notification =
  bind_rows(df_events_notification_resent,
            df_events_notification_not_resent)

# Subset the events packets to those with notification dates and
# combinations of individual-level characteristics when only a single
# such contact was notified that day.
df_events_notification_unique = inner_join(
  df_events_notification,
```

```
    df_analytics_notified_unique,
    by = join_by(date_notified, ltla, postcode_district, os_version,
                 device_model)
)

# Assign an arbitrary identifier (an integer) to each such
# combination, indicating one contact.
df_events_notification_unique = df_events_notification_unique %>%
  mutate(.by = c(date_notified, ltla, postcode_district, os_version,
         device_model),
         contact_id = cur_group_id()) %>%
  arrange(contact_id)

# Exclude days of exposure for which a single putative contact had more
# than 48 exposure windows (very rare).
df_events_notification_unique = df_events_notification_unique %>%
  filter(.by = c(contact_id, date_exposure),
         n() <= 48)

# Use the reported-positive outcome for each event packet to define a
# reported-positive outcome for each putative contact. If a contact has
# only one event packet, we use its positivity outcome; if they have
# at least two packets, they are positive if at least two of their
# packets are positive.
df_events_notification_unique = df_events_notification_unique %>%
  mutate(.by = contact_id,
         positive_contact = ifelse(n() == 1,
                                   unique(positive_packet),
                                   sum(positive_packet) >= 2))
```
